# Supplementary material for: Pharmacogenomics-assisted schizophrenia management: A hybrid type 2 effectiveness-implementation study protocol to compare the clinical utility, cost-effectiveness, and barriers
Source: PLoS One. 2024 Apr 10;19(4):e0300511. doi: 10.1371/journal.pone.0300511 (PMC11006179; doi:10.1371/journal.pone.0300511)
Supplement: S2 File — (DOCX) [file pone.0300511.s002.docx]

**Protocol TEMPLATE: Clinical Trials**

| Complete Title | Clinical utility and cost-effectiveness of pharmacogenomics-assisted treatment versus standard of care in patients with schizophrenia attending a tertiary care hospital in eastern India | |
| --- | --- | --- |
| Short Title | Clinical utility and cost-effectiveness of pharmacogenomics-assisted treatment in schizophrenia | |
| Protocol Identification no. | ICMR-CAM/SRUM/2023-24/01 | |
| Principal Investigator | Dr. Saibal Das, Scientist D (Medical), ICMR-Centre for Ageing and Mental Health, Block: DP-1, Sector V, Salt Lake, Kolkata: 700 091 | |
| Protocol Date and version | 11/05/2023, v 6.0 | |
| Amendment 1 Date: | | Amendment 3 Date: |
| Amendment 2 Date: | | Amendment 4 Date: |

**Principal Investigator with complete postal address, Mobile No., Telephone/ Fax No. and Email**

- Dr. Saibal Das, Scientist D (Medical), ICMR-Centre for Ageing and Mental Health, Block: DP-1, Sector V, Salt Lake, Kolkata: 700 091, Tel: 8072486512, Email: [saibaldas123@gmail.com](mailto:saibaldas123@gmail.com)

**Co-Investigator(s) with complete postal address, Mobile No., Telephone/ Fax No. and Email**

- Dr. Aniruddha Basu, Associate Professor, Department of Psychiatry, AIIMS, Kalyani,  NH - 34 Connector Basantapur, Kalyani, West Bengal 741245, Tel: 8968979239, Email: [aniruddha.psy@aiimskalyani.edu.in](mailto:aniruddha.psy@aiimskalyani.edu.in)
- Dr. Kumari Rina, Assistant Professor, Department of Psychiatry, AIIMS, Kalyani,  NH - 34 Connector Basantapur, Kalyani, West Bengal 741245, Tel: 9914576444, Email: [drkumaririna@gmail.com](mailto:drkumaririna@gmail.com)
- Dr. Sukanto Sarkar, Additional Professor, Department of Psychiatry, AIIMS, Kalyani,  NH - 34 Connector Basantapur, Kalyani, West Bengal 741245, Tel: 9655489210, Email: [sukanto.psy@aiimskalyani.edu.in](mailto:sukanto.psy@aiimskalyani.edu.in)
- Dr. Atanu Kumar Dutta, Additional. Professor, Department of Biochemistry, AIIMS, Kalyani,  NH - 34 Connector Basantapur, Kalyani, West Bengal 741245, Tel: 8967369455, Email: [atanu.biochem@aiimskalyani.edu.in](mailto:atanu.biochem@aiimskalyani.edu.in)
- Dr. Deepasree Sukumaran, Assistant Professor, Department of Pharmacology, AIIMS, Kalyani,  NH - 34 Connector Basantapur, Kalyani, West Bengal 741245, Tel: 9626587446, Email: [deepasree.sukumaran@gmail.com](mailto:deepasree.sukumaran@gmail.com)
- Dr. Bhavani Shankara Bagepally, Scientist E, ICMR-National Institute of Epidemiology, Chennai, Tel: 9980574800, Email: [bshankara@gmail.com](mailto:bshankara@gmail.com)
- Dr. Pawan Kumar Maurya, Scientist B, ICMR-Centre for Ageing and Mental Health, Block: DP-1, Sector V, Salt Lake, Kolkata: 700 091, Tel: 9908023634, Email: [maurya_pawan@yahoo.com](mailto:maurya_pawan@yahoo.com)
- Dr. Manoj Kalita, Scientist C, ICMR-Centre for Ageing and Mental Health, Block: DP-1, Sector V, Salt Lake, Kolkata: 700 091, Tel: 8099133633, Email: kalita.manoj@icmr.gov.in
- Dr. Indranil Saha, Scientist E (Medical), ICMR-Centre for Ageing and Mental Health, Block: DP-1, Sector V, Salt Lake, Kolkata: 700 091, Tel: 9830019016, Email: [drsahaindranil@gmail.com](mailto:drsahaindranil@gmail.com%20)

**Abbreviations/Acronyms**

| **Abbreviation** | **Full word / term** |
| --- | --- |
| AEs | Adverse events |
| DSM V | Diagnostic and statistical manual V |
| PGx | Pharmacogenomics |
| RCT | Randomized controlled trial |
| SAEs | Serious adverse events |
| SOC | Standard of care |
| SNP | Single nucleotide polymorphisms |

**One Page Protocol summary**

**Title:** Clinical utility and cost-effectiveness of pharmacogenomics-assisted treatment versus standard of care in patients with schizophrenia attending a tertiary care hospital in eastern India

**Background:** The typical and atypical antipsychotics derive their therapeutic benefit predominantly from the antagonism of dopamine D2 and 5-HT2A receptors. Many of these compounds are associated with common and significant adverse effects (e.g. weight gain, extrapyramidal symptoms, hyperprolactinemia, sexual dysfunction, and cardiac effects), which negatively impact adherence.

**Novelty:** To date, no randomized controlled trial (RCT) has been conducted to evaluate the outcomes in patients taking antipsychotics with treatments assisted/guided by pharmacogenomics (PGx). The use of PGx information as an interventional tool to guide antipsychotic treatment is the study novelty (hybrid effectiveness-implementation design type 2 study).

**Aim:** To evaluate the clinical utility, cost-effectiveness, and feasibility of pharmacogenomics-assisted treatment versus standard of care (SOC) in patients with schizophrenia attending a tertiary care hospital in eastern India.

**Methods:** In part 1, an RCT will be performed. Adult patients diagnosed with schizophrenia (DSM V) will be randomized (2: 1) into two arms administered to receive PGx-assisted therapy (drug and dose selection depending on the results of the following nine SNPs in genes *DRD2, HTR1A, HTR2C, CYP2D6, CYP3A5, CYP1A2, and CYP3A4*) or SOC. Serum drug levels will be estimated and correlated with dosing and clinical response. The patients will be followed up for 12 weeks. The two groups will be compared for the reduction in the incidence of solicited AEs and SAEs, requirement and duration of hospitalization, medication adherence, time to clinical response, number and olanzapine-equivalent doses of various anti-psychotics, and quality of life. In part 2, the cost-effectiveness analysis of PGx-assisted treatment as compared to SOC will be evaluated in the same patients. In part 3, the facilitators and challenges in implementing PGx-assisted treatment for schizophrenia will be explored in a qualitative study.

**Expected outcome:** The study findings will help in understanding whether PGx-assisted treatment has a clinical utility or is cost-effective while treating patients with schizophrenia. This study is a hybrid type 2 effectiveness-implementation research.

**Keywords:** schizophrenia, anti-psychotics, schizophrenia, pharmacogenomics, therapeutic drug monitoring, cost-effectiveness.

**Protocol Synopsis**

| Study Title | Clinical utility and cost-effectiveness of pharmacogenomics-assisted treatment versus standard of care in patients with schizophrenia attending a tertiary care hospital in eastern India |
| --- | --- |
| Type of study | **Part 1:** Randomized controlled trial  **Part 2:** Pharmacoeconomics study  **Part 3:** Qualitative study |
| Study Rationale | According to the National Mental Health Survey of India 2015–2016, the prevalence of psychotic disorders in India is ~1.5%. Response to antipsychotic therapy is highly variable, and it is not possible to predict those patients who will or will not respond to medications. Furthermore, around 30% of these patients are treatment-resistant. The typical and atypical antipsychotics derive their therapeutic benefit predominantly from the antagonism of dopamine D2 and 5-HT2A receptors. Many of these compounds are associated with common and significant adverse effects (e.g. weight gain, extrapyramidal symptoms, hyperprolactinemia, sexual dysfunction, and cardiac effects), which negatively impact adherence. Multiple studies have investigated pharmacogenomics (PGx) approaches to identify genotype-specific dosing and predict antipsychotic responses and/or adverse effects. Medication optimization interventions (e.g. PGx-assisted treatment) based on the concept of precision medicine in people on polypharmacy due to psychotic disorders are complex and limited; a more holistic and integrated approach is warranted. PGx testing can be seen as a companion decision-support tool, under consideration of all relevant individual clinical and demographic information available. The cost-effectiveness of such interventions is also unclear. To date, no randomized controlled trial (RCT) has been conducted to evaluate the outcomes in patients taking antipsychotics with treatments assisted/guided by PGx. The use of PGx information as an interventional tool to guide antipsychotic treatment is the study novelty (hybrid effectiveness-implementation design type 2 study). |
| Study Objective(s) | - To evaluate the clinical utility (safety and efficacy) of PGx-assisted treatment as compared to standard of care in terms of reduction in the incidence of solicited AEs and SAEs, requirement and duration of hospitalization, and time to respond in patients with schizophrenia. - To estimate the cost-effectiveness of PGx-assisted treatment as compared to the standard of care in patients with schizophrenia. - To explore the facilitators and challenges in implementing PGx-assisted treatment for schizophrenia. |
| Intervention | PGx-assisted treatment |
| Indication | Schizophrenia (DSM V) |
| Study Design | In part 1, an RCT will be performed. Adult patients diagnosed with schizophrenia (DSM V) will be randomized (2: 1) into two arms administered to receive PGx-assisted therapy (drug and dose selection depending on the results of the following nine SNPs in genes *DRD2, HTR1A, HTR2C, CYP2D6, CYP3A5, CYP1A2, and CYP3A4*) or SOC. Serum drug levels will be estimated and correlated with dosing and clinical response. The patients will be followed up for 12 weeks. The two groups will be compared for the reduction in the incidence of solicited AEs and SAEs, requirement and duration of hospitalization, medication adherence, time to clinical response, number and olanzapine-equivalent doses of various anti-psychotics, and quality of life. In part 2, the cost-effectiveness analysis of PGx-assisted treatment as compared to SOC will be evaluated in the same patients. In part 3, the facilitators and challenges in implementing PGx-assisted treatment for schizophrenia will be explored in a qualitative study. |
| Subject Population  key criteria for Inclusion and Exclusion: | - **Inclusion criteria:** Adult (≥18 years) patients of both gender attending the OPD of Psychiatry, AIIMS, Kalyani, diagnosed with schizophrenia (DSM V), and are adjudicated by the treating psychiatrist to be treated with one or more of the following first-line antipsychotic medicine: olanzapine, risperidone, haloperidol, amisulpride, quetiapine, aripiprazole, and trifluoperazine. - **Exclusion criteria:** Patients who are needed to be hospitalized or provided electroconvulsive therapy (ECT) at presentation, patients for whom any other antipsychotic drug is required to be initiated, and those who are unwilling to provide informed consent. |
| Number of Participants | 250 |
| Study Duration | Each subject’s participation will last for 12 weeks.  The entire study is expected to last for 3 years. |
| Study Phases | In part 1, an RCT will be performed. Adult patients diagnosed with schizophrenia (DSM V) will be randomized (2: 1) into two arms administered to receive PGx-assisted therapy (drug and dose selection depending on the results of the following nine SNPs in genes *DRD2, HTR1A, HTR2C, CYP2D6, CYP3A5, CYP1A2, and CYP3A4*) or SOC. Serum drug levels will be estimated and correlated with dosing and clinical response. The patients will be followed up for 12 weeks. The two groups will be compared for the reduction in the incidence of solicited AEs and SAEs, requirement and duration of hospitalization, medication adherence, time to clinical response, number and olanzapine-equivalent doses of various anti-psychotics, and quality of life. In part 2, the cost-effectiveness analysis of PGx-assisted treatment as compared to SOC will be evaluated in the same patients. In part 3, the facilitators and challenges in implementing PGx-assisted treatment for schizophrenia will be explored in a qualitative study. |
| Primary endpoint | The difference in the Udvalg for Kliniske Undersøgelser Side-Effect Rating Scale (UKU-SERS) score between the two groups |
| Secondary endpoints | - The proportion of patients developing solicited AEs and SAEs - The proportion of patients requiring hospitalization - Duration of hospitalization - Clinical response at week 12 (PANSS and CGI) - Time-to-achieve clinical response through 12 weeks (PANSS and CGI) - The proportion of patients non-responsive to treatment - Number and olanzapine-equivalent doses of various anti-psychotics - Medication adherence - Quality of life (EQ-5D-5L) - Correlation between serum drug level, dosing, and clinical response - Health outcomes in terms of life years, QALYs, and costs - Facilitators and challenges in implementing PGx-assisted treatment |
| Statistical And Analytic Plan | - **Part 1:** The data will be checked for normal distribution (Kolmogorov-Smirnov test). For categorical variables (e.g. response rates, safety outcomes) the chi-squared test will be used. For continuous variables (e.g. scale scores, duration of hospitalization), analysis of covariance (ANCOVA) with treatment in the model and baseline clinical and demographic characteristics and drug dosage as covariates will be used. Subgroup analysis will be performed based on the treatment-status (treatment naïve and who received prior treatment), educational level, negative symptoms from first psychotic episode, PANSS and CGI scores, comorbid substance use; age at onset, lack of early response, and adherence to treatment. The Kaplan-Meier estimate will be used to draw the survival curves denoting the time-to-achieve response. Patients will be labeled responsive if they have a ≥20% reduction in PANSS score at the end of 6 weeks as compared to the score at baseline.^46^ Cox's proportional hazards model will be used to assess the difference in the time to respond between the two groups allowing for other covariates. For correlation between drug dosing, drug levels, and clinical response Pearson’s or Spearman correlation test will be applied. A p-value of <0.05 will be considered significant. Interim analyses will be performed after the follow-up of 90, 180, and 270 patients. O'Brien-Fleming boundary will be used for stopping. - **Part 2:** We will develop a decision analytic model from the health system’s perspective from the patients’ data using a short-term decision tree and a long-term (lifetime horizon) Markov model. - **Part 3:** A thematic analysis will be performed. |
| Safety Evaluations | UKU-SERS and SMARTS questionnaire |
| Data and Safety Monitoring Plan | The patients will be monitored by the treating physicians for clinical efficacy and safety. DSMB will be constituted if advised by the Institutional Ethics Committee. |

# 1. Background Information and Rationale

## Introduction

# According to the National Mental Health Survey of India 2015–2016, the prevalence of psychotic disorders in India is ~1.5%.^1^ Response to antipsychotic therapy is highly variable, and it is not possible to predict those patients who will or will not respond to medications. Furthermore, around 30% of these patients are treatment-resistant.^2^ Across several studies, it has been found that the proportion of patients receiving antipsychotic polypharmacy ranged from 15.9–60.5% before they received clozapine (treatment-resistant schizophrenia).^3^ Treatment of these patients imposes a huge burden on the patient and the health system. The typical and atypical antipsychotics derive their therapeutic benefit predominantly from the antagonism of dopamine D2 and 5-HT2A receptors. Many of these compounds are associated with common and significant adverse effects (e.g. weight gain, extrapyramidal symptoms, hyperprolactinemia, sexual dysfunction, and cardiac effects), which negatively impact adherence. Pharmacogenomic (PGx) factors play a major role in deciding treatment responses to antipsychotic medicines.^2^

Multiple studies have investigated PGx approaches to identify genotype-specific dosing and predict antipsychotic responses and/or adverse effects.^4^ Currently, the US FDA provides information on pharmacogenomic biomarkers in their drug labeling for nine antipsychotics (aripiprazole, aripiprazole lauroxil, brexpiprazole, clozapine, iloperidone, perphenazine, pimozide, risperidone, and thioridazine).^5^ Seven of nine refer to CYP2D6 PM status, where dose adjustment recommendations are provided for the following antipsychotics: aripiprazole, aripiprazole lauroxil,brexpiprazole, clozapine, iloperidone, pimozide, and thioridazine. Similarly, Pharmacogenomics Knowledgebase (PharmGKB) website lists ten antipsychotics where caution is advised for patients who are poor CYP2D6 metabolizers.^6^ Drug labels with PGx information are provided for the following antipsychotics: aripiprazole, aripiprazole lauroxil, brexpiprazole, clozapine, iloperidone, olanzapine, perphenazine, pimozide, risperidone, and thioridazine. The Royal Dutch Association for the Advancement of Pharmacy-Dutch Pharmacogenetics Working Group has provided PGx drug dosing guidelines based on CYP2D6 genotypes for six antipsychotics: aripiprazole, clozapine, haloperidol, olanzapine, risperidone, and zuclopenthixol.^7^ However, some studies showed negative results.^8^

Medication optimization interventions (e.g. PGx-assisted treatment) based on the concept of precision medicine in people on polypharmacy due to psychotic disorders are complex and limited; a more holistic and integrated approach is warranted.^9^ PGx testing can be seen as a companion decision-support tool, under consideration of all relevant individual clinical and demographic information available. The cost-effectiveness of such interventions is also unclear.^10^ The main goal of precision medicine in neuropsychiatric conditions is to use genetic and brain-imaging information to improve safety, efficacy, and outcomes. The role of genetic factors in determining the response to antipsychotic treatment has been researched. Theoretically, genes that code for proteins involved in a drug’s pharmacodynamics and pharmacokinetics could affect the therapeutic response and safety. The vast majority of common variants associated with treatment response and adverse effects of antipsychotics were identified only in the samples of European ancestry. The associated variants identified in populations of European ancestry might not be significant in other ancestry groups, because of underlying genetic heterogeneity.

The efficacy of PGx-guided treatment in patients with depression has been demonstrated in several randomized controlled trials and meta-analyses. The findings indicate that treatment guided by PGx testing, including combinatorial testing, is superior to treatment as usual in response and/or remission rates in the acute treatment of depression.^2^ However, to date, no randomized controlled study (RCT) has been conducted to evaluate the outcomes in patients taking antipsychotics following the use of PGx guidance of treatment selections.

## Novelty

Although there are several observational studies that aimed to find associations between genetic variants and treatment response (efficacy and safety), there is no study that had used PGx-assisted intervention (selection of antipsychotic drugs and their doses based on genotype) to optimize treatment in patients with schizophrenia. The use of PGx information as an interventional tool to guide treatment is the novelty of this study. PGx information will be correlated with antipsychotic drug levels (therapeutic drug monitoring), dosing, and clinical response. Further, the cost-effectiveness of PGx-assisted treatment in schizophrenia has not been evaluated in India. Finally, facilitators and challenges in implementing PGx-assisted treatment will be also explored using a qualitative study. Hence, this is a hybrid type 2 effectiveness-implementation research.

## Findings from previous studies

**Table 1.** Association between genetic polymorphisms and antipsychotic serum (plasma) concentrations or dosage.

|  | Study | | Gene(s) | Polymorphism(s) | Study design | Subjects, *n* | Ethnicity or | Treatment | Treatment | Outcome | Main findings |  |
| --- | --- | --- | --- | --- | --- | --- | --- | --- | --- | --- | --- | --- |
|  | (year) | |  |  |  |  | nationality | duration | medication |  |  |  |
|  |  |  |  |  |  |  |  |  |  |  |  |  |
|  | Hettige | | 29 candidate | 109 SNPs | Cross-sectional | 263 SCZ | European | Not reported | Various | Antipsychotic | Significant association |  |
|  | et al. | | genes |  |  |  |  |  | antipsychotic | dosage | between *GABRB1* |  |
| (2016) | | |  |  |  |  |  |  | drugs |  | (rs16860087 and rs4627835) |  |
|  |  |  |  |  |  |  |  |  |  |  | and higher antipsychotic |  |
|  |  |  |  |  |  |  |  |  |  |  | dosage |  |
|  |  | |  |  |  |  |  |  |  |  |  |  |
|  |  | Hettige | Exploratory | Exploratory | GWAS | 83 SCZ and SAD | Caucasian, | Not reported | Various | Antipsychotic | Polygenic risk score |  |
|  | et al. | |  |  |  |  | African, |  | antipsychotic | dosage | revealed no significant |  |
| (2016) | | |  |  |  |  | Asian |  | drugs |  | association with |  |
|  |  |  |  |  |  |  |  |  |  |  | antipsychotic dosage |  |
|  |  | |  |  |  |  |  |  |  |  |  |  |
|  |  | Koga | Exploratory | Exploratory | GWAS | 79 SCZ and SAD | Caucasian | Not reported | Mainly olanzapine, | Antipsychotic | No SNPs were associated |  |
|  | et al. | |  |  |  |  |  |  | risperidone, | dosage | with antipsychotic dosage at |  |
| (2016) | | |  |  |  |  |  |  | clozapine |  | a genome-wide level |  |
|  | | |  |  |  |  |  |  |  |  |  |  |
|  | Piatkov | | *ABCB1* | rs1045642 | Prospective | 137 SCZ | Caucasian, | 12 months | Clozapine | Clozapine and | No significant association; |  |
| et al. | | | *ABCC1* | rs212090 |  |  | Asian, Pacific |  |  | norclozapine | the combination of *ABCB1* |  |
| (2017) | | |  |  |  |  | Islander, |  |  | concentrations | and *ABCC1* homozygote |  |
|  |  |  |  |  |  |  | others |  |  |  | SNPs was associated with |  |
|  |  |  |  |  |  |  |  |  |  |  | increased clozapine and |  |
|  |  |  |  |  |  |  |  |  |  |  | norclozapine serum levels |  |
|  | | |  |  |  |  |  |  |  |  |  |  |
|  | | van der Weide | *CYP3A4* | *CYP3A4*22* | Retrospective | 834 psychotic | Mainly | 1,380 days | Aripiprazole, | Antipsychotic | *CYP2D6* genotype affected |  |
| and van der | | | *CYP2D6* | *CYP2D6* |  | disorders | Caucasian | on average | haloperidol, | concentration | dose-corrected |  |
| Weide | | |  | polymorphisms |  |  |  |  | pimozide, |  | concentrations of the |  |
| (2015) | | |  |  |  |  |  |  | risperidone |  | antipsychotics |  |
|  | | |  |  |  |  |  |  |  |  |  |  |
| Czerwensky | | | *CYP1A2* | *CYP1A2*1D,* | Naturalistic | 98 SCZ, | Caucasian | ≥4 weeks | Olanzapine | Olanzapine | Significant association |  |
| et al. | | | *UGT1A4* | *CYP1A2*1F* | and | schizotypal, | except for |  | (monotherapy or | concentration | between *CYP1A2*1D* and |  |
| (2015) | | |  | *UGT1A4*3* | retrospective | paranoid | 1 Asian |  | in combination |  | *CYP1A2*1F* polymorphisms |  |
|  |  |  | *POR* | rs2302429 |  | disorder, mood |  |  | with other |  | and olanzapine serum |  |
|  |  |  |  |  |  | disorders, |  |  | antipsychotics) |  | concentration |  |
|  |  |  |  |  |  | affective |  |  |  |  |  |  |
|  |  |  |  |  |  | disorder, and |  |  |  |  |  |  |
|  |  |  |  |  |  | other disorders |  |  |  |  |  |  |
|  |  |  |  |  |  | treated with |  |  |  |  |  |  |
|  |  |  |  |  |  | SGAs |  |  |  |  |  |  |
|  | |  |  |  |  |  |  |  |  |  |  |  |
| Cabaleiro | | | Several | Several candidate | Randomized | 79 healthy | Caucasian, | 24 h | Quetiapine | Quetiapine | Pharmacokinetics was |  |
| et al. | | | candidate | polymorphisms | cross-over |  | except for 2 |  |  | concentration | affected by *DRD3* and |  |
| (2015) | | | genes |  |  |  | (from |  |  |  | *CYP1A2* polymorphisms, |  |
|  |  |  |  |  |  |  | Guatemala) |  |  |  | and pharmacodynamics was |  |
|  |  |  |  |  |  |  |  |  |  |  | affected by *CYP2C19* |  |
|  |  |  |  |  |  |  |  |  |  |  | polymorphisms |  |

**Table 2.** Association between genetic polymorphisms and clinical response to antipsychotic medications

| Study |  | Gene(s) | Polymorphism(s) | Study design | Subjects, *n* | Ethnicity or | Treatment duration | Treatment medication | Outcome | Main findings |  |  |  |
| --- | --- | --- | --- | --- | --- | --- | --- | --- | --- | --- | --- | --- | --- |
| (year) |  |  |  |  |  | nationality |  |  |  |  |  |  |  |
|  |  |  |  |  |  |  |  |  |  |  |  |  |  |
| McGregor | *MMP9* | | Several SNPs | Prospective | 103 FES | South African, | 12 months | Flupenthixol decanoate | PANSS | Several variants within *MMP9* showed | | |  |
| et al. |  |  |  |  |  | Xhosa, Caucasian |  |  |  | associations with treatment response |  |  |  |
| (2018) |  |  |  |  |  |  |  |  |  |  |  |  |  |
|  |  |  |  |  |  |  |  |  |  |  |  |  |  |
| Sun | *COMT* | | rs165599, rs4680 | Prospective | 96 SCZ | Han Chinese | 8 weeks | Various antipsychotic drugs | PANSS | Significant association between *COMT* rs4680 | | |  |
| et al. |  |  |  |  |  |  |  |  |  | and treatment response |  |  |  |
| (2018) |  |  |  |  |  |  |  |  |  |  |  |  |  |
|  |  |  |  |  |  |  |  |  |  |  |  |  |  |
| Yu | Exploratory | | Exploratory | GWAS | Discovery sample: | Discovery sample: | Sample A: 6 weeks; | Discovery sample: aripipra- | PANSS | Five novel loci (rs72790443 in *MEGF10*, | | |  |
| et al. |  |  |  |  | 2,413 SCZ; Replication | Han Chinese; | Sample B: 8 weeks | zole, olanzapine, quetiapine, |  | rs1471786 in *SLC1A1*, rs9291547 in *PCDH7*, |  |  |  |
| (2018) |  |  |  |  | sample: 1,379 SCZ | Replication sample: |  | risperidone, ziprasidone, or |  | rs12711680 in *CNTNAP5*, and rs6444970 in |  |  |  |
|  |  |  |  |  |  | Han Chinese |  | one of the firstgeneration­ |  | *TNIK*) were significantly associated with |  |  |  |
|  |  |  |  |  |  |  |  | antipsychotics (haloperidol or |  | treatment response; furthermore, three |  |  |  |
|  |  |  |  |  |  |  |  | perphenazine); Replication |  | additional loci were associated with drug- |  |  |  |
|  |  |  |  |  |  |  |  | sample: olanzapine, risperi- |  | specific treatment responses (rs2239063 in |  |  |  |
|  |  |  |  |  |  |  |  | done, or aripiprazole |  | *CANCA1C* for olanzapine, rs16921385 in |  |  |  |
|  |  |  |  |  |  |  |  |  |  | *SLC1A1* for risperidone, and rs17022006 in |  |  |  |
|  |  |  |  |  |  |  |  |  |  | *CNTN4* for aripiprazole) |  |  |  |
|  |  |  |  |  |  |  |  |  |  |  |  |  |  |
| Calabrò | *COMT*, *GSK3B*, *HTR2A*, | | 12 SNPs | Naturalistic | 83 SCZ | Italian | 10 days | Various antipsychotic drugs | PANSS | Suggested association between *PLA2G4A* | | |  |
| et al. | *PLA2G4A*, *SIGMAR1* | |  |  |  |  |  |  |  | rs1214459 and positive symptom response | | |  |
| (2018) |  |  |  |  |  |  |  |  |  |  |  |  |  |
|  | *CHRNA7*, *COMT*, *CREB1*, | | 49 SNPs | Naturalistic | 176 SCZ | Korean | 37.5±17.08 days | Olanzapine, quetiapine, | PANSS | Suggested associations between *CHRNA7* | | |  |
|  |  |  |  |  |  |  |  |  |  |  |  |  |  |
|  | *GSK3B*, *HTR2A*, *MAPK1*, | |  |  |  |  |  | risperidone |  | (rs11071511), *HTR2A* (rs1328685), and | | |  |
|  | *PLA2G4A*, *S100B*, *SIG-* | |  |  |  |  |  |  |  | *PLA2G4A* (rs10798069) and positive symptom | | |  |
|  | *MAR1* | |  |  |  |  |  |  |  | response; suggested associations between | | |  |
|  |  |  |  |  |  |  |  |  |  | *CHRNA7* (rs2337980 and rs6494223), *HTR2A* |  |  |  |
|  |  |  |  |  |  |  |  |  |  | (rs643627), and *SIGMAR1* (rs10814130) and |  |  |  |
|  |  |  |  |  |  |  |  |  |  | negative symptom response |  |  |  |
|  |  | |  |  |  |  |  |  |  |  | | |  |
| Li | Exploratory | | Exploratory | GWAS, Sample A: | Sample A: 171 SCZ; | Sample A: | Sample A: 6 weeks; | Lurasidone | PANSS | No significant findings after multiple testing | | |  |
| et al. |  |  |  | double-blind, | Sample B: 131 SCZ | Caucasian; | Sample B: 6 weeks |  |  | correction; common genetic variants related |  |  |  |
| (2018) |  |  |  | randomized; Sample |  | Sample B: African |  |  |  | to synaptic adhesion complexes, scaffolding, |  |  |  |
|  |  |  |  | B: double-blind, |  | American |  |  |  | and the alternative splicing regulator may be |  |  |  |
|  |  |  |  | randomized |  |  |  |  |  | associated with treatment response to lurasi- |  |  |  |
|  |  |  |  |  |  |  |  |  |  | done |  |  |  |
|  |  | |  |  |  |  |  |  |  |  | | |  |
| Calabrò | *BDNF*, *ESYT2*, *NCAPG2*, | | 15 SNPs | Naturalistic | 83 SCZ | Italian | 10 days | Various antipsychotic drugs | PANSS | Suggested associations between *ESYT2* | | |  |
| et al. | *PKDCC*, *VIPR2*, *WDR60* | |  |  |  |  |  |  |  | rs2657375 and positive symptom response | | |  |
| (2017) |  |  |  |  |  |  |  |  |  |  |  |  |  |
|  | *ARC*, *BDNF*, *CHL1*, *ESYT2*, 51 SNPs | | | Naturalistic | 176 SCZ | Korean | 37.5±17.08 days | Olanzapine, quetiapine, | PANSS | *HOMER1* rs3822568 was nominally associated |  |  |  |
|  |  |  |  |  |  |  |  |  |  |  |  |  |  |
|  | *HOMER1*, *NCAPG2*, | |  |  |  |  |  | risperidone |  | with antipsychotic response; suggested |  |  |  |
|  | *TXNRD2*, *VIPR2*, *WDR60* | |  |  |  |  |  |  |  | associations between *TXNRD2* rs4646310 and |  |  |  |
|  |  |  |  |  |  |  |  |  |  | positive symptom response; suggested |  |  |  |
|  |  |  |  |  |  |  |  |  |  | association between *ARC* rs10110456 and |  |  |  |
|  |  |  |  |  |  |  |  |  |  | *HOMER1* rs6893883 and negative symptom |  |  |  |
|  |  |  |  |  |  |  |  |  |  | response |  |  |  |
|  |  | |  |  |  |  |  |  |  |  | | |  |
| Kang | *SNAP25* | | rs8636, rs3746544 | Observational | 101 SCZ | Korean | 6 weeks | Amisulpride | PANSS | Significant association between *SNAP25* | | |  |
| et al. |  |  |  |  |  |  |  |  |  | rs8636 and treatment response |  |  |  |
| (2017) |  |  |  |  |  |  |  |  |  |  |  |  |  |
|  |  | |  |  |  |  |  |  |  |  | | |  |
| Kaur | *DRD2* | | rs1800497, | Prospective | SCZ | Indian | 12 weeks | Risperidone | PANSS | Significant associations between *DRD2* | | |  |
| et al. |  |  | rs1079597, |  |  |  |  |  |  | rs180498 and *HTR2A* rs6305 and treatment |  |  |  |
| (2017) |  |  | rs180498, |  |  |  |  |  |  | response; *CYP2D6* rs3892097 was significantly |  |  |  |
|  |  |  | rs1801028 |  |  |  |  |  |  | associated with treatment response when |  |  |  |
|  | *HTR2A* | | rs6313, rs6311, |  |  |  |  |  |  | dropouts were excluded from analysis | | |  |
|  |  |  | rs6305 |  |  |  |  |  |  |  |  |  |  |
|  | *CYP2D6* | | rs3892097, rs106585 |  |  |  |  |  |  |  | | |  |
|  |  | |  |  |  |  |  |  |  |  | | |  |
| Papazisis | *ABCB1* | | rs2032582, rs1045642 | Naturalistic | SCZ or other psychotic | Caucasian | 4 weeks | Various antipsychotic drugs | PANSS | No significant association; however, a combi- | | |  |
| et al. |  |  | common variants |  | disorder |  |  |  |  | nation of a loss-of-function *CYP2D6* allele and |  |  |  |
| (2018) | *CYP2D6* | |  |  |  |  |  |  |  | the TT genotype of *ABCB1* rs2032582 were | | |  |
|  |  |  |  |  |  |  |  |  |  | associated with poor response to antipsychotic |  |  |  |
|  |  |  |  |  |  |  |  |  |  | treatment |  |  |  |
|  |  | |  |  |  |  |  |  |  |  | | |  |
| Shi | 100 risperidone response- | | Exploratory | Prospective | 288 SCZ | Chinese | 4 weeks | Risperidone | PANSS | *UGT1A3* rs6706232 and *COMT* rs4818 were | | |  |
| et al. | related genes | |  |  |  |  |  |  |  | significantly associated with treatment | | |  |
| (2017) |  |  |  |  |  |  |  |  |  | response in the meta-analysis |  |  |  |
|  |  |  |  |  |  |  |  |  |  |  |  |  |  |

|  |  |  |  |  |  |  |  |  |  |  |  |  |  |  |  |  |  |  |  |  |  |  |  |  |  |
| --- | --- | --- | --- | --- | --- | --- | --- | --- | --- | --- | --- | --- | --- | --- | --- | --- | --- | --- | --- | --- | --- | --- | --- | --- | --- |
|  |  |  |  |  |  |  |  |  |  |  |  |  |  |  |  |  |  |  |  |  |  |  |  |  |  |
|  | | | | | |  |  | | |  | |  |  | | |  | |  | | |  | | |  |  |
| Kang | | | | | | *ANKS1B* | rs7968606 | | | Prospective | | 101 SCZ | Korean | | | 6 weeks | | Amisulpride | | | PANSS | | | Significant association between *ANKS1B* |  |
| et al. | | | | | |  |  | | |  | |  |  | | |  | |  | | |  | | | rs7968606 and treatment response |  |
| (2017) | | | | | |  |  | | |  | |  |  | | |  | |  | | |  | | |  |  |
|  | | | | | |  |  | | |  | |  |  | | |  | |  | | |  | | |  |  |
| Taylor | | | | | | *SLC1A2* | rs4354668, | | | Prospective | | 163 SCZ and SAD | European | | | 6 months | | Clozapine | | | BPRS | | | No significant association |  |
| et al. | | | | | |  | rs4534557, | | |  | |  |  | | |  | |  | | |  | | |  |  |
| (2017) | | | | | | *SLC6A9* | rs2901534 | | |  | |  |  | | |  | |  | | |  | | |  |  |
|  | | | | | |  | rs12037805, | | |  | |  |  | | |  | |  | | |  | | |  |  |
|  | | | | | |  | rs1978195, | | |  | |  |  | | |  | |  | | |  | | |  |  |
|  | | | | | |  | rs16831558 | | |  | |  |  | | |  | |  | | |  | | |  |  |
|  | | | | | | *GRIA1* | rs2195450 | | |  | |  |  | | |  | |  | | |  | | |  |  |
|  | | | | | | *GRM2* | rs4067, | | |  | |  |  | | |  | |  | | |  | | |  |  |
|  | | | | | |  | rs2518461 | | |  | |  |  | | |  | |  | | |  | | |  |  |
|  | | | | | | *GAD1* | rs3749034 | | |  | |  |  | | |  | |  | | |  | | |  |  |
|  | | | | | |  |  | | |  | |  |  | | |  | |  | | |  | | |  |  |
| Ovenden | | | | | | Exploratory | Exploratory | | | GWAS, prospective | | 103 FES | South African | | | 12 months | | Flupenthixol decanoate | | | PANSS | | | Suggested associations between *MANBA*, |  |
| et al. | | | | | |  |  | | |  | |  |  | | |  | |  | | |  | | | *COL9A2*, and *NFKB1* and treatment response |  |
| (2017) | | | | | |  |  | | |  | |  |  | | |  | |  | | |  | | |  |  |
|  | | | | | |  |  | | |  | |  |  | | |  | |  | | |  | | |  |  |
| Li | | | | | | Exploratory | Exploratory | | | GWAS | | 1,390 SCZ from 12 | European | | | ≥6 weeks | | Paliperidone extended-release | | | PANSS | | | *ADCK1* gene variants may be associated with |  |
| et al. | | | | | |  |  | | |  | | trials |  | | |  | | or paliperidone palmitate | | |  | | | paliperidone efficacy |  |
| (2017) | | | | | |  |  | | |  | |  |  | | |  | |  | | |  | | |  |  |
|  | | | | | |  |  | | |  | |  |  | | |  | |  | | |  | | |  |  |
| Sacchetti | | | | | | Exploratory | Exploratory | | | GWAS | | Discovery sample: 86 | Sample A: Cauca- | | | Sample A: 2 weeks; | | Risperidone | | | PANSS | | | Significant association between *GRM7* |  |
| et al. | | | | | |  |  | | |  | | SCZ; Replication | sian; Sample B: | | | Sample B: 9 months | |  | | |  | | | rs2133450 and treatment response |  |
| (2017) | | | | | |  |  | | |  | | sample: 97 SCZ, SAD | European | | |  | |  | | |  | | |  |  |
|  | | | | | |  |  | | |  | |  |  | | |  | |  | | |  | | |  |  |
| Labad | | | | | | *ESR1* | rs9340799, rs2234693, | | | Double-blind, | | 65 SCZ (female, | Spanish | | | 24 weeks | | Raloxifene | | | PANSS | | | *UGT1A8* rs1042597 and *ESR1* rs2234693 were |  |
| et al. | | | | | |  | rs1801132 | | | randomized | | postmenopausal |  | | |  | |  | | |  | | | significantly associated with negative symp- |  |
| (2016) | | | | | |  | rs1042597 | | |  | | status) |  | | |  | |  | | |  | | | toms and general psychopathology, respec- |  |
|  | | | | | | *UGT1A8* |  | | |  | |  |  | | |  | |  | | |  | | | tively |  |
|  | | | | | |  |  | | |  | |  |  | | |  | |  | | |  | | |  |  |
| Brandl | | | | | | *ITIH3* | rs2535629 | | | Sample A: prospec- | | 256 SCZ, SAD | European, African | | | Sample A: up to 6 | | Sample A: various antipsy- | | | BPRS | | | Significant association of *ITIH3* rs2535629 |  |
| et al. | | | | | |  |  | | | tive; Sample B: | |  | American | | | weeks; Sample B: up | | chotic drugs; Sample B: | | |  | | | with improvement of negative symptoms in |  |
| (2016) | | | | | |  |  | | | prospective; Sample | |  |  | | | to 6 months; Sample | | clozapine; Sample C: various | | |  | | | Europeans |  |
|  | | | | | |  |  | | | C: double-blind, | |  |  | | | C: up to 14 weeks; | | antipsychotic drugs; Sample | | |  | | |  |  |
|  | | | | | |  |  | | | randomized; Sample | |  |  | | | Sample D: up to 6 | | D: clozapine | | |  | | |  |  |
|  | | | | | |  |  | | | D: prospective | |  |  | | | months | |  | | |  | | |  |  |
|  | | | | | |  |  | | |  | |  |  | | |  | |  | | |  | | |  |  |
| Terzić | | | | | | *DRD1* | rs4532, rs5326 | | | Case-control | | 138 SCZ, 94 healthy | Slovenian | | | Not reported | | Various antipsychotic drugs | | | PANSS, | | | No significant association between any of the |  |
| et al. | | | | | | *DRD2* | rs1801028, | | |  | |  |  | | |  | |  | | | BPRS, and | | | genotypes and treatment response, and the oc- |  |
| (2016) | | | | | | *DRD3* | rs1799732 | | |  | |  |  | | |  | |  | | | GAF | | | currence of treatment-resistant schizophrenia |  |
|  | | | | | |  | rs6280 | | |  | |  |  | | |  | |  | | |  | | |  |  |
|  | | | | | | *COMT* | rs165815, rs4680 | | |  | |  |  | | |  | |  | | |  | | |  |  |
|  | | | | | |  |  | | |  | |  |  | | |  | |  | | |  | | |  |  |
| Porcelli | | | | | | *PDE7B* | rs975676, | | | Case-control | | 573 SCZ, 560 healthy | Korean | | | 20–40 days | | Various antipsychotic drugs | | | PANSS | | | Significant association between *EPM2A* |  |
| et al. | | | | | |  | rs3734548 | | |  | |  |  | | |  | |  | | |  | | | rs1415744 and treatment response in negative |  |
| (2016) | | | | | | *NMBR* | rs2717, rs6926279, | | |  | |  |  | | |  | |  | | |  | | | symptoms |  |
|  | | | | | |  | rs6902780 | | |  | |  |  | | |  | |  | | |  | | |  |  |
|  | | | | | |  | rs1415744, | | |  | |  |  | | |  | |  | | |  | | |  |  |
|  | | | | | | *EPM2A* | rs702304, rs2235481 | | |  | |  |  | | |  | |  | | |  | | |  |  |
|  | | | | | |  |  | | |  | |  |  | | |  | |  | | |  | | |  |  |
| Drögemöller | | | | | | Exploratory | Exploratory | | | Whole-exome | | Discovery sample: 103 | Discovery sample: | | | Discovery sample: 3 | | Discovery sample: flupenthix- | | | Discovery | | | Significant association between rs13025959 |  |
| et al. | | | | | |  |  | | | sequencing | | FES; Replication | South African | | | months; Replication | | ol decanoate; Replication | | | sample: | | | in *MYO7B* (E1647D) and rs10380 in *MTRR* |  |
| (2016) | | | | | |  |  | | |  | | sample: 87 FES | Colored, Xhosa, | | | sample: ≥3 months | | sample: various antipsychotic | | | PANSS; | | | (H622Y) and treatment response |  |
|  | | | | | |  |  | | |  | |  | European; Replica- | | |  | | drugs | | | Replication | | |  |  |
|  | | | | | |  |  | | |  | |  | tion sample: | | |  | |  | | | sample: BPRS | | |  |  |
|  | | | | | |  |  | | |  | |  | African American, | | |  | |  | | |  | | |  |  |
|  | | | | | |  |  | | |  | |  | European | | |  | |  | | |  | | |  |  |
|  | | | | | |  |  | | |  | |  |  | | |  | |  | | |  | | |  |  |
| Stevenson | | | | | Candidate genes involved | | | | Candidate SNPs and | GWAS, prospective | Discovery sample: 86 | | Discovery sample: | | Discovery sample: | | Discovery sample: various | | | BPRS | | Significant association between *GRM7* | | |  |
| et al. | | | | | with glutamate signaling | | | | exploratory | (open-label) | first-episode patients | | Caucasian, African | | 6 weeks | | antipsychotic drugs (mainly | | |  | | (rs2069062 and rs2014195) in candidate gene | | |  |
| (2016) | | | | | and exploratory | | | |  |  | (SCZ, SAD, psychotic | | American | |  | | risperidone monotherapy); | | |  | | analysis and *GRID2* (rs9307122 and | | |  |
|  | | | | |  | | | |  |  | bipolar disorder, or | |  | |  | | Replication sample: risperi- | | |  | | rs1875705) in GWAS analysis and treatment | | |  |
|  | | | | |  | | | |  |  | major depressive | |  | |  | | done | | |  | | response | | |  |
|  | | | | |  | | | |  |  | disorder with psychot- | |  | |  | |  | | |  | |  | | |  |
|  | | | | |  | | | |  |  | ic features); Replica- | |  | |  | |  | | |  | |  | | |  |
|  | | | | |  | | | |  |  | tion sample: 240 | |  | |  | |  | | |  | |  | | |  |
|  | | | | |  | | | |  |  | patients from the | |  | |  | |  | | |  | |  | | |  |
|  | | | | |  | | | |  |  | CATIE study | |  | |  | |  | | |  | |  | | |  |
|  | | | | |  | | | |  |  |  | |  | |  | |  | | |  | |  | | |  |
| Taylor | | | | | *GRIN2B* | | | | rs3764030, rs7301328, | Prospective | 175 SCZ and SAD | | European | | 6 months | | Clozapine | | | BPRS | | No significant association | | |  |
| et al. | | | | |  | | | | rs12826365, rs1072388, |  |  | |  | |  | |  | | |  | |  | | |  |
| (2016) | | | | |  | | | | rs2284411, rs1806201, |  |  | |  | |  | |  | | |  | |  | | |  |
|  | | | | |  | | | | rs1806191, rs890 |  |  | |  | |  | |  | | |  | |  | | |  |
|  | | | | |  | | | |  |  |  | |  | |  | |  | | |  | |  | | |  |
| Jajodia | | | | | Candidate genes of NRG1- | | | | 1,536 SNPs | Cross-sectional | 742 SCZ and 786 | | Indian | | 3 months | | Various antipsychotic drugs | | | CGI | | *CCL2* (rs4795893, rs4586) and *GRIA4* | | |  |
| et al. | | | | | ERBB signaling pathway, | | | |  | and naturalistic | healthy | |  | |  | |  | | |  | | (rs2513265) in the low severity group, and | | |  |
| (2016) | | | | | neuroactive ligand-receptor | | | |  |  |  | |  | |  | |  | | |  | | *ADCY2* (rs1544938) and *NRG1* (rs13250975 | | |  |
|  | | | | | interaction, glutamate | | | |  |  |  | |  | |  | |  | | |  | | and rs17716295) in the high severity group | | |  |
|  | | | | | signaling | | | |  |  |  | |  | |  | |  | | |  | | were associated with treatment response | | |  |
|  | | | | |  | | | |  |  |  | |  | |  | |  | | |  | |  | | |  |
| Chen | | | | | *COMT* | | | | rs2075507, rs737865, | Naturalistic | 185 SCZ | | Han Chinese | | 12 weeks | | Amisulpride | | | PANSS | | Significant association between *COMT* | | |  |
| et al. | | | | |  | | | | rs933271, rs5993883, |  |  | |  | |  | |  | | |  | | variants (rs4680, rs4633, and rs6267) and | | |  |
| (2016) | | | | |  | | | | rs740603, rs4646312, |  |  | |  | |  | |  | | |  | | treatment response, particularly negative | | |  |
|  | | | | |  | | | | rs4633, rs6267, rs4818, |  |  | |  | |  | |  | | |  | | symptoms | | |  |
|  | | | | |  | | | | rs4680, rs165774, |  |  | |  | |  | |  | | |  | |  | | |  |
|  | | | | |  | | | | rs174697, rs165599, |  |  | |  | |  | |  | | |  | |  | | |  |
|  | | | | |  | | | | rs165728 |  |  | |  | |  | |  | | |  | |  | | |  |
|  | | | | |  | | | |  |  |  | |  | |  | |  | | |  | |  | | |  |
| Huang | | | | | *DRD2* | | | | rs2514218 | Prospective | 208 SCZ | | Caucasian, African | | 6 months | | Clozapine | | | BPRS | | Significant association between *DRD2* | | |  |
| et al. | | | | |  | | | |  |  |  | | American, and | |  | |  | | |  | | rs2514218 and treatment response | | |  |
| (2016) | | | | |  | | | |  |  |  | | others | |  | |  | | |  | |  | | |  |
|  | | | | |  | | | |  |  |  | |  | |  | |  | | |  | |  | | |  |
| Xu | | | | | 25 candidate genes includ- | | | | 77 SNPs | Prospective | 995 SCZ | | Han Chinese | | 2 weeks | | Risperidone, clozapine, | | | PANSS | | Significant associations of several genes, | | |  |
| et al. | | | | | ing *CYP2D6*, *CYP2C19*, | | | |  |  |  | |  | |  | | quetiapine, chlorpromazine | | |  | | including new candidate genes, with treat- | | |  |
| (2016) | | | | | *COMT*, *ABCB1*, *DRD3*, and | | | |  |  |  | |  | |  | |  | | |  | | ment response; however, most of the associa- | | |  |
|  | | | | | *HTR2C* | | | |  |  |  | |  | |  | |  | | |  | | tions remained no longer after multiple | | |  |
|  | | | | |  | | | |  |  |  | |  | |  | |  | | |  | | corrections, except for *COMT* (rs6269, | | |  |
|  | | | | |  | | | |  |  |  | |  | |  | |  | | |  | | rs5993883, and rs4818); significant association | | |  |
|  | | | | |  | | | |  |  |  | |  | |  | |  | | |  | | of haplotype rs1544325-rs5993883-rs6269- | | |  |
|  | | | | |  | | | |  |  |  | |  | |  | |  | | |  | | rs4818 in *COMT* with treatment response; the | | |  |
|  | | | | |  | | | |  |  |  | |  | |  | |  | | |  | | combination of rs6269 in *COMT* and | | |  |
|  | | | | |  | | | |  |  |  | |  | |  | |  | | |  | | rs3813929 in *HTR2C* may be associated with | | |  |
|  | | | | |  | | | |  |  |  | |  | |  | |  | | |  | | treatment response | | |  |
|  | | | | |  | | | |  |  |  | |  | |  | |  | | |  | |  | | |  |
| Takekita | | | | | *HTR7* | | | | rs12412496, | Randomized | 100 SCZ | | Japanese | | 12 weeks | | Aripiprazole or perospirone | | | PANSS | | No significant association | | |  |
| et al. | | | | |  | | | | rs7916403, | open-label |  | |  | |  | |  | | |  | |  | | |  |
| (2015) | | | | |  | | | | rs1935349 |  |  | |  | |  | |  | | |  | |  | | |  |
|  | | | | |  | | | |  |  |  | |  | |  | |  | | |  | |  | | |  |
| Lee | | | | | *ALDH2* | | | | *ALDH2* polymor- | Double-blind, | 149 SCZ | | Taiwanese | | 11 weeks | | Risperidone + dextrome­ | | | PANSS, | | The *ALDH2*2*2* genotype was significantly | | |  |
| et al. | | | | |  | | | | phisms | randomized |  | |  | |  | | thorphan or risperidone + | | | SANS | | associated with negative symptoms in the | | |  |
| (2015) | | | | |  | | | |  |  |  | |  | |  | | placebo | | |  | | risperidone + dextromethorphan group | | |  |
|  | | | | |  | | | |  |  |  | |  | |  | |  | | |  | |  | | |  |
| Zhang | | | | | *DRD2* | | | | rs2514218 | Double-blind, | 198 FEP | | Asian, African | | 12 weeks | | Aripiprazole or risperidone | | | BPRS, | | Homozygotes for the rs2514218 risk allele (C) | | |  |
| et al. | | | | |  | | | |  | randomized |  | | American, Hispan- | |  | |  | | | SANS | | had a significantly greater reduction in | | |  |
| (2015) | | | | |  | | | |  |  |  | | ic, Caucasian, and | |  | |  | | |  | | positive symptoms than T-allele carriers | | |  |
|  | | | | |  | | | |  |  |  | | others | |  | |  | | |  | |  | | |  |
|  | | | | |  | | | |  |  |  | |  | |  | |  | | |  | |  | | |  |
| Le Clerc | | | | | Exploratory | | | | Exploratory | GWAS, prospective | 89 SCZ | | Caucasian | | 6 weeks | | Olanzapine or risperidone | | | BPRS, | | Significant association between *PPP1R18* | | |  |
| et al. | | | | |  | | | |  |  |  | |  | |  | |  | | | PANSS | | rs3129996 and treatment response | | |  |
| (2015) | | | | |  | | | |  |  |  | |  | |  | |  | | |  | |  | | |  |
|  | | | | |  | | | |  |  |  | |  | |  | |  | | |  | |  | | |  |
| Porcelli | | | | | *CACNA1C* | | | | 24 SNPs | Case-control | 176 SCZ, 326 healthy | | Korean | | Not reported | | Risperidone, olanzapine, | | | PANSS | | Five SNPs (rs723672, rs1034936, rs2283271, | | |  |
| et al. | | | | |  | | | |  |  |  | |  | |  | | quetiapine | | |  | | rs10848635, and rs1016388) were associated | | |  |
| (2015) | | | | |  | | | |  |  |  | |  | |  | |  | | |  | | with an improvement in PANSS scores | | |  |
|  | | | | |  | | | |  |  |  | |  | |  | |  | | |  | |  | | |  |
|  | | | | |  | | | |  |  |  | |  | |  | |  | | |  | |  | | |  |
|  |  |  |  |  |  |  |  |  |  |  |  |  |  |  |  |  |  |  |  |  |  |  |  |  |  |
|  |  |  |  |  |  |  |  |  |  |  |  |  |  |  |  |  |  |  |  |  |  |  |  |  |  |
|  |  |  |  | |  | | |  | |  | |  | |  | |  | | |  | |  | |  | |  |
|  |  | Takekita | | | *HTR1A* | | | rs6295, rs1364043, | | Randomized | | 100 SCZ | | Japanese | | 12 weeks | | | Aripiprazole or perospirone | | PANSS | | *HTR1A* rs1364043 and the rs10042486- | |  |
|  |  | et al. | | |  | | | rs878567, rs10042486 | | open-label | |  | |  | |  | | |  | |  | | rs6295-rs1364043 haplotype may affect | |  |
| (2015) | | | | |  | | |  | |  | |  | |  | |  | | |  | |  | | negative symptoms in PANSS | |  |
|  |  |  |  | |  | | |  | |  | |  | |  | |  | | |  | |  | |  | |  |
|  |  | Terzic | | | *5-HT1A* | | | rs6295 | | Case-control | | 138 SCZ (94 treatment | | Slovenian | | Not reported | | | Various antipsychotic drugs | | PANSS, | | Association between *5-HT1A* rs6295 and GAF | |  |
|  |  | et al. | | | *SLC6A4* | | | *5-HTTLPR* | |  | | responsive and 44 | |  | |  | | | including clozapine | | BPRS, CGI, | | score; association between the three-allelic | |  |
| (2015) | | | | |  | | |  | |  | | treatment resistant) | |  | |  | | |  | | GAF | | *5-HTTLPR* polymorphism and GAF score and | |  |
|  |  |  |  | |  | | |  | |  | | and 94 healthy | |  | |  | | |  | |  | | the negative subscale score of the PANSS | |  |
|  |  |  |  | |  | | |  | |  | |  | |  | |  | | |  | |  | |  | |  |
|  |  | Pai | | | *GRIN1* | | | rs11146020 | | Prospective | | 195 SCZ and 136 | | Indian | | 12 weeks | | | Mainly risperidone and | | CGI-S | | No significant association | |  |
|  |  | et al. | | | *ABCB1* | | | rs1045642, | |  | | healthy | |  | |  | | | olanzapine | |  | |  | |  |
| (2015) | | | | | *DRD4* | | | rs2032582 | |  | |  | |  | |  | | |  | |  | |  | |  |
|  |  |  |  | |  |  |  | rs1800955, | |  | |  | |  | |  | | |  | |  | |  | |  |
|  |  |  |  | |  | | | rs4646984 | |  | |  | |  | |  | | |  | |  | |  | |  |
|  |  |  |  | |  | | |  | |  | |  | |  | |  | | |  | |  | |  | |  |
|  |  | Wang | | | Exploratory | | | Exploratory | | GWAS | | Discovery sample: 684 | |  | | Discovery sample: 6, | | | Paliperidone | | PANSS | | *ERBB4* rs6435681 was significantly associated | |  |
|  |  | et al. | | |  | | |  | |  | | SCZ; Replication | |  | | 9, or 30 weeks; | | |  | |  | | with treatment response | |  |
| (2015) | | | | |  | | |  | |  | | sample: 2,856 SCZ | |  | | Replication sample: | | |  | |  | |  | |  |
|  |  |  |  | |  | | |  | |  | |  | |  | | 2, 6, 13, or 53 weeks | | |  | |  | |  | |  |
|  |  |  |  | |  | | |  | |  | |  | |  | |  | | |  | |  | |  | |  |
|  |  | Kang | | | *DRD2* | | | rs1079597, rs1800497 | | Prospective | | 125 SCZ | | Korean | | 6 weeks | | | Amisulpride | | PANSS | | Significant association between *DRD2* | |  |
|  |  | et al | | |  | | |  | |  | |  | |  | |  | | |  | |  | | rs1079597 and treatment response | |  |
| (2015) | | | | |  | | |  | |  | |  | |  | |  | | |  | |  | |  | |  |
|  |  |  | | |  | | |  | | | |  | |  | |  | | |  | |  | |  | |  |
|  |  | Porcelli | | | *AHI1* | | | rs11154801, rs7750586, Case-control | | | | 426 SCZ and 345 | | Korean | | Not reported | | | Not reported | | PANSS | | *AHI1* rs7750586 and rs9647635 were | |  |
|  |  | et al. | | |  | | | rs9647635, rs9321501 | |  | | healthy | |  | |  | | |  | |  | | associated with treatment response of | |  |
| (2015) | | | | |  | | |  | |  | |  | |  | |  | | |  | |  | | negative symptoms | |  |
|  |  |  | | |  | | |  | |  | |  | |  | |  | | |  | |  | |  | |  |
|  |  | Blasi | | | *DRD2* | | | rs1076560 | | Sample A: prospec- | | Sample A: 63 SCZ; | | Caucasian | | Sample A: 8 weeks; | | | Sample A: olanzapine; Sample | | PANSS | | *DRD2* rs1076560 and *HTR2A* rs6314 together | |  |
|  |  | et al. | | | *HTR2A* | | | rs6314 | | tive; Sample B: | | Sample B: 54 SCZ, 620 | |  | | Sample B: 4 weeks | | | B: various antipsychotic drugs | |  | | affected the treatment response | |  |
| (2015) | | | | |  | | |  | | double-blind, | | healthy | |  | |  | | |  | |  | |  | |  |
|  |  |  |  | |  | | |  | | randomized | |  | |  | |  | | |  | |  | |  | |  |
|  |  |  |  | |  | | |  | |  | |  | |  | |  | | |  | |  | |  | |  |
|  |  |  | Bosia | | *COMT* | | | rs4680 | | Prospective | | 107 SCZ | | Italian | | ≥3 months | | | Clozapine | | PANSS | | Significant association between *COMT* and | |  |
|  |  | et al. | | | *5-HT1A-R* | | | rs6295 | |  | |  | |  | |  | | |  | |  | | *5-HT1A-R* and treatment response in negative | |  |
| (2015) | | | | |  | | |  | |  | |  | |  | |  | | |  | |  | | symptom variation | |  |
|  |  |  | | |  | | |  | |  | | | |  | |  | | |  | |  | |  | |  |
|  |  | Pouget | | | *TSPO* | | | rs739092, rs5759197, | | Sample A: naturalis- 161 SCZ and SAD | | | | European | | Up to 14 weeks | | | Clozapine, olanzapine, | | BPRS | | No significant association | |  |
|  | et al. | | | |  | | | rs138911, rs113515, | | tic; Sample B: | |  | |  | |  | | | quetiapine, risperidone, | |  | |  | |  |
| (2015) | | | | |  | | | rs6971/rs6973, | | prospective; Sample | |  | |  | |  | | | others | |  | |  | |  |
|  |  |  |  | |  | | | rs80411, rs138926 | | C: double-blind, | |  | |  | |  | | |  | |  | |  | |  |
|  |  |  |  | |  | | |  | | randomized; Sample | |  | |  | |  | | |  | |  | |  | |  |
|  |  |  |  | |  | | |  | | D: prospective | |  | |  | |  | | |  | |  | |  | |  |
|  |  | |  | |  | | |  | |  | |  | |  | |  | | |  | |  | |  | |  |
|  |  |  | Huo | | *DRD1* | | | rs5326, rs4867798, | | Prospective | | 185 SCZ | | Han Chinese | | 4 weeks | | | Risperidone | | PANSS | | No significant association | |  |
|  | et al. | | | |  | | | rs4532, rs686 | |  | |  | |  | |  | | |  | |  | |  | |  |
| (2015) | | | | |  | | |  | |  | |  | |  | |  | | |  | |  | |  | |  |
|  |  | | | |  | | |  | |  | |  | |  | |  | | |  | |  | |  | |  |
|  | Brandl | | | | *CYP3A43* | | | rs472660, rs680055 | | Prospective | | 152 SCZ or SAD | | European | | Up to 6 months | | | Various antipsychotics | | BPRS | | Significant association between *CYP3A43* | |  |
|  | et al | | | |  | | |  | |  | | (Sample A: 86; | |  | |  | | | including clozapine | |  | | rs680055 and treatment response | |  |
| (2015) | | | | |  | | |  | |  | | Sample B: 66) | |  | |  | | |  | |  | |  | |  |
|  |  | | | |  | | |  | |  | |  | |  | |  | | |  | |  | |  | |  |
|  | Bishop | | | | *GRM3* | | | rs6465084, rs274622, | | Prospective | | 61 untreated FES, | | Caucasian, African | | 6 weeks | | | Mainly risperidone | | BPRS | | Negative symptom improvement was | |  |
| et al | | | | |  | | | rs1989796, rs1468412, | |  | | SAD, schizophreni- | | American, Hispan- | |  | | |  | |  | | associated with rs6465084 in the *GRM3* | |  |
| (2015) | | | | |  | | | rs2228595 | |  | | form disorder; 130 | | ic, and Asian | |  | | |  | |  | | gene | |  |
|  |  |  |  | | *DRD2/ANKK1* | | | rs1799732 | |  | | healthy | |  | |  | | |  | |  | |  | |  |
|  |  |  |  | |  | | | (−141C | |  | |  | |  | |  | | |  | |  | |  | |  |
|  |  |  |  | |  | | | Ins/Del), | |  | |  | |  | |  | | |  | |  | |  | |  |
|  |  |  |  | |  | | | rs1800497 | |  | |  | |  | |  | | |  | |  | |  | |  |
|  |  |  |  | |  | | | (TaqIA) | |  | |  | |  | |  | | |  | |  | |  | |  |
|  |  |  |  | | *COMT* | | | rs4680 (Val158Met) | |  | |  | |  | |  | | |  | |  | |  | |  |
|  | | |  | |  | | |  | |  | |  | |  | |  | | |  | |  | |  | |  |
|  |  |  |  |  |  |  |  |  |  |  |  |  |  |  |  |  |  |  |  |  |  |  |  |  |  |
|  |  |  |  |  |  |  |  |  |  |  |  |  |  |  |  |  |  |  |  |  |  |  |  |  |  |
|  |  |  |  |  |  |  |  |  |  |  |  |  |  |  |  |  |  |  |  |  |  |  |  |  |  |
|  |  |  |  |  |  |  |  |  |  |  |  |  |  |  |  |  |  |  |  |  |  |  |  |  |  |
|  |  |  |  |  |  |  |  |  |  |  |  |  |  |  |  |  |  |  |  |  |  |  |  |  |  |

**Table 3.** Association between genetic polymorphisms and antipsychotic adverse effects.

| Study | Gene(s) | Polymor- | Study design | Subjects, *n* | Ethnicity or | Treatment duration | Treatment medication | Outcome | Main findings |
| --- | --- | --- | --- | --- | --- | --- | --- | --- | --- |
| (year) |  | phism(s) |  |  | nationality |  |  |  |  |
|  | | |  |  |  |  |  |  |  |
| **Antipsychotic-induced weight gain and metabolic syndrome** | | |  |  |  |  |  |  |  |
| Mittal | mtDNA and 670 | mtDNA SNPs | mtDNA sequenc- | 74 SCZ and 168 SCZ | Caucasian | 143±11.98 days | Risperidone, quetiapine, | AIWG | Thirty nuclear-encoded mitochon- |
| et al. | nuclear-encoded | and nuclear- | ing and gene set |  |  |  | olanzapine |  | drial genes were nominally signifi- |
| (2017) | mitochondrial | encoded | analysis |  |  |  |  |  | cantly associated with AIWG; of |
|  | genes | mitochondrial |  |  |  |  |  |  | these, the associations of three genes |
|  |  | genes |  |  |  |  |  |  | (*CLPB*, *PARL*, and *ACAD10*) with |
|  |  |  |  |  |  |  |  |  | AIWG were replicated |
|  |  |  |  |  |  |  |  |  |  |
| Piatkov | *ABCB1* | rs1045642 | Prospective | 137 SCZ | Caucasian, Asian, | 12 months | Clozapine | AIWG | No significant association; *ABCB1* |
| et al. | *ABCC1* | rs212090 |  |  | Pacific Islander, |  |  |  | rs1045642 and *ABCC1* rs212090 were |
| (2017) |  |  |  |  | others |  |  |  | associated with AIWG in males after |
|  |  |  |  |  |  |  |  |  | 3 months and 12 months of clozapine |
|  |  |  |  |  |  |  |  |  | treatment, respectively |
|  |  |  |  |  |  |  |  |  |  |
| Li | 23 genes | 43 SNPs | Prospective | 339 SCZ and SAD (86 | Chinese | 12 weeks | Olanzapine, risperidone, | AIWG | *TOX* rs11777927 and *ADIPOQ* |
| et al. |  |  |  | first-episode patients) |  |  | clozapine, quetiapine, |  | rs182052 were associated with BMI; |
| (2017) |  |  |  |  |  |  | aripiprazole, ziprasidone |  | *BDNF* rs6265, *BDAF* rs11030104, and |
|  |  |  |  |  |  |  |  |  | *ADIPOQ* rs822396 were significantly |
|  |  |  |  |  |  |  |  |  | associated with a change in waist-to- |
|  |  |  |  |  |  |  |  |  | hip ratio |
|  |  |  |  |  |  |  |  |  |  |
| Klemettilä | 21 genes (*NPY* | 215 SNPs | Cross-sectional | 180 SCZ (F2 group | Finnish | ≥3 months (some | Clozapine | AIWG | 21 genes (*NPY* gene, *NPY* receptor |
| et al. | gene, *NPY* |  |  | according to ICD-10) |  | patients with no |  |  | genes, and genes encoding arcuate |
| (2017) | receptor genes, |  |  |  |  | data) |  |  | nucleus NPY neuron receptors) |
|  | and genes |  |  |  |  |  |  |  |  |
|  | encoding |  |  |  |  |  |  |  |  |
|  | arcuate nucleus |  |  |  |  |  |  |  |  |
|  | NPY neuron |  |  |  |  |  |  |  |  |
|  | receptors) |  |  |  |  |  |  |  |  |
|  |  |  |  |  |  |  |  |  |  |
| Daray | *HTR2C* | rs3813939 | Prospective | 48 SCZ or related illness | Caucasian | 6 weeks | Risperidone, olanzapine, | AIWG | T allele at position −759 (TT or CT) |
| et al. |  | (−759C>T) |  | (female) |  |  | clozapine, quetiapine |  | was associated with less weight gain |
| (2017) |  |  |  |  |  |  |  |  |  |
|  |  |  |  |  |  |  |  |  |  |
| Zhang | *C3* | 3 tag SNPs and | Cross-sectional | 576 SCZ | Han Chinese | 2 years | Clozapine monotherapy | MetS | Significant association between *C3* |
| et al. |  | rs7951, |  |  |  |  | or combination therapy |  | rs2277984 and MetS |
| (2017) |  | rs2230199, |  |  |  |  |  |  |  |
|  |  | rs2250656, |  |  |  |  |  |  |  |
|  |  | rs11672613 |  |  |  |  |  |  |  |
|  |  |  |  |  |  |  |  |  |  |
| Koskinen | *INSIG2* | rs1559509, | Cross-sectional | 190 SCZ (F2 group | Finnish | ≥3 months | Clozapine | AIWG | Significant association between |
| et al. |  | rs2161829, |  | according to ICD-10) |  |  |  |  | *INSIG2* SNPs (rs12151787, rs1049626, |
| (2016) |  | rs2161830, |  |  |  |  |  |  | and rs17047733) and weight gain |
|  |  | rs9308762, |  |  |  |  |  |  |  |
|  |  | rs12151787, |  |  |  |  |  |  |  |
|  |  | rs1049626, |  |  |  |  |  |  |  |
|  |  | rs17047733 |  |  |  |  |  |  |  |
|  |  |  |  |  |  |  |  |  |  |
| Brandl | Exploratory | Exploratory | GWAS | CATIE sample: 189 SCZ; | CATIE sample: | CATIE sample: up to | CATIE sample: risperi- | AIWG | None of the SNPs were significantly |
| et al. |  |  |  | Toronto sample: 86 SCZ | European or African | 18 months; Toronto | done, quetiapine, olan- |  | associated with AIWG, although |
| (2016) |  |  |  | and SAD | American; Toronto | sample: up to 6 weeks | zapine; Toronto sample: |  | nominal associations were found for |
|  |  |  |  |  | sample: European | (Sample A), 6 weeks | olanzapine, clozapine |  | rs9346455 upstream of *OGFRL1* and |
|  |  |  |  |  |  | (Sample B), or 14 |  |  | rs1059778 in *IBA57* |
|  |  |  |  |  |  | weeks |  |  |  |
|  |  |  |  |  |  |  |  |  |  |
| Zai | *HTR3A* | 21 SNPs | Sample A: natural- | 149 SCZ and SAD | European | Sample A: up to | Sample A: haloperidol | AIWG | No significant association |
| et al. | *HTR3B* |  | istic; Sample B: |  |  | 6 weeks; Sample B: | olanzapine, risperidone, |  |  |
| (2016) |  |  | prospective; |  |  | 6 weeks; Sample C: | aripiprazole, quetiapine, |  |  |
|  |  |  | Sample C: double- |  |  | up to 14 weeks | amisulpride; Sample B: |  |  |
|  |  |  | blind, randomized |  |  |  | clozapine; Sample C: |  |  |
|  |  |  |  |  |  |  | clozapine, olanzapine |  |  |
|  |  |  |  |  |  |  |  |  |  |
| Grădinaru | *HTR2C* | rs3813929 | Prospective | 81 SCZ and bipolar | Romanian | Up to 18 months | Risperidone, aripiprazole, | AIWG and | No significant association between |
| et al. |  | (−759C/T) |  | disorder |  |  | olanzapine | hyperinsu- | *HTR2C* rs3813929 and AIWG; |
| (2016) |  |  |  |  |  |  |  | linemia | however, a significant association |
|  |  |  |  |  |  |  |  |  | between insulinemia and T-allele |
|  |  |  |  |  |  |  |  |  | carriers was found |
|  |  |  |  |  |  |  |  |  |  |

|  |  |  |  |  |  |  |  |  |  |
| --- | --- | --- | --- | --- | --- | --- | --- | --- | --- |
| Tiwari | *HRH1* | 40 tag SNPs | Retrospective | 193 SCZ and SAD | European American, | Up to 14 weeks | Clozapine, haloperidol, | AIWG | No significant associations between |
| et al. | *HRH3* |  |  |  | African American, |  | olanzapine, risperidone, |  | the SNPs in *HRH*1 and *HRH3* and |
| (2016) |  |  |  |  | and others |  | and others |  | AIWG |
|  |  |  |  |  |  |  |  |  |  |
| Fang | *BDNF* | rs6265 | Cross-sectional | 308 chronic schizophrenia, | Han Chinese | ≥12 months | Clozapine, risperidone, | AIWG | Significant association between *BDNF* |
| et al. |  | (Val66Met) |  | 304 healthy |  |  | other typical antipsychot- |  | rs6265 and AIWG |
| (2016) |  |  |  |  |  |  | ics |  |  |
|  |  |  |  |  |  |  |  |  |  |
| Rico-Gomis | *HTR2C* | rs1414334 | Cross-sectional, | 166 SCZ, SAD, schizo- | Spanish | ≥3 months | Various antipsychotics | MetS | No significant association between |
| et al. |  |  | observational | phreniform disorder, other |  |  |  |  | the C allele of the rs1414334 polymor- |
| (2016) |  |  |  | psychotic disorders, |  |  |  |  | phism in the *HTR2C* gene and MetS |
|  |  |  |  | bipolar disorder |  |  |  |  |  |
|  |  |  |  |  |  |  |  |  |  |
| Yang | *SCAP* | 5 SNPs | Cross-sectional | 722 SCZ | Han Chinese | ≥1 year | Clozapine, olanzapine, | MetS | Significant association between the |
| et al. | *SREBF1* | 11SNPs |  |  |  |  | risperidone |  | rs11654081 T allele of the *SREBF1* |
| (2016) |  |  |  |  |  |  |  |  | gene and an increased risk for MetS |
|  |  |  |  |  |  |  |  |  |  |
| Ryu | 60 candidate | 233 SNPs | Prospective | Sample A: 84 SCZ; Sample | Korean | Sample A: up to 8 | Sample A: risperidone, | AIWG and | No significant association between |
| et al. | genes |  |  | B: 46 SCZ |  | weeks; Sample B: | olanzapine, amisulpride, | appetite | these SNPs and BMI or appetite |
| (2016) |  |  |  |  |  | ≥4 weeks (up to 48 | quetiapine, and others; | change | change; *GHRL* rs696217 suggested |
|  |  |  |  |  |  | weeks) | Sample B: risperidone, |  | evidence of an association with |
|  |  |  |  |  |  |  | olanzapine |  | weight gain and appetite change |
|  |  |  |  |  |  |  |  |  |  |
| Yu | Exploratory | Exploratory | GWAS | Discovery sample: 534 | Han Chinese | Discovery sample: | Various antipsychotics | AIWG | Significant association between |
| et al. |  |  |  | SCZ; Replication sample: |  | 8 weeks; Replication |  |  | rs10977144 and rs10977154 in |
| (2016) |  |  |  | 547 SCZ |  | sample: 8 weeks |  |  | *PTPRD* and rs12386481 in *GFPT2* |
|  |  |  |  |  |  |  |  |  | and AIWG |
|  |  |  |  |  |  |  |  |  |  |
| Tiwari | *HCRTR1* | Tag SNPs | Sample A: natural- | Discovery sample: 218 SCZ | Discovery sample: | Discovery sample: up | Discovery sample: | AIWG | Genetic variation in *HCRTR2* was |
| et al. | *HCRTR2* | (exploratory) | istic; Sample B: | and SAD (Sample A: *n* = | European American, | to 6 weeks (Sample | clozapine, haloperidol, |  | associated with weight gain; none of |
| (2016) |  |  | prospective; | 88; Sample B: *n* = 74; | African American, | A), 6 weeks (Sample | olanzapine, risperidone, |  | the SNPs in *HCRTR*1 were associated |
|  |  |  | Sample C: double- | Sample C: *n* = 56); Replica- | and others; Replica- | B), or 14 weeks (Sam- | and others (Sample A); |  | with weight gain |
|  |  |  | blind, randomized | tion sample: 122 SCZ | tion sample: Euro- | ple C); Replication | clozapine (Sample B); |  |  |
|  |  |  |  |  | pean | sample: up to 190 | clozapine, haloperidol, |  |  |
|  |  |  |  |  |  | days | olanzapine, risperidone |  |  |
|  |  |  |  |  |  |  | (Sample C); Replication |  |  |
|  |  |  |  |  |  |  | sample: olanzapine, |  |  |
|  |  |  |  |  |  |  | risperidone |  |  |
|  |  |  |  |  |  |  |  |  |  |
| Wang | 85 candidate | 768 SNPs | Prospective | 216 SCZ | Han Chinese | 4 weeks | Risperidone | AIWG | Significant association between four |
| et al. | genes |  |  |  |  |  |  |  | SNPs on *SLC6A4* (rs3813034, |
| (2015) |  |  |  |  |  |  |  |  | rs1042173, rs4325622, and rs9303628) |
|  |  |  |  |  |  |  |  |  | and AIWG |
|  |  |  |  |  |  |  |  |  |  |
| Chen | *TMEM18* | rs6548238 | Double-blind, | 55 SCZ, SAD (metformin | Taiwanese | 24 weeks | Clozapine | AIWG | Significant association in the |
| et al. | *SH2B1* | rs7498665 | randomized | treatment [*n* = 28] or |  |  |  |  | *TMEM18* and *GNPDA2* minor-allele |
| (2015) | *GNPDA2* | rs10938397 |  | placebo [*n* = 27]) |  |  |  |  | carrier groups; body weight reduction |
|  |  |  |  |  |  |  |  |  | in the metformin group |
|  |  |  |  |  |  |  |  |  |  |
| Dong | *A2BP1* | rs10500331, | Prospective | Discovery sample: 328 | Han Chinese | Discovery sample: | Olanzapine | AIWG | Significant association between |
| et al. |  | rs4786847, |  | SCZ; Replication sample: |  | 8 weeks; Replication |  |  | *A2BP1* rs1478697 and AIWG |
| (2015) |  | rs8048076, |  | 208 FES and drug-naïve |  | sample: 4 weeks |  |  |  |
|  |  | rs1478697 |  | SCZ |  |  |  |  |  |
|  |  |  |  |  |  |  |  |  |  |
| Bonaccorso | *BDNF* | rs6265 | Randomized trial, | 76 SCZ and SAD; 90 | Caucasian, African | 12 months | Olanzapine, risperidone | AIWG, | Significant association between *BDNF* |
| et al. |  | (Val66Met) | prospective | bipolar disorder | American, others |  |  | metabolic | Met66 allele carriers and AIWG in all |
| (2015) |  |  |  |  |  |  |  | parameters | patients |
|  |  |  |  |  |  |  |  |  |  |

## Relevant Literature and Data

# According to the National Mental Health Survey of India 2015–2016, the prevalence of psychotic disorders in India is ~1.5%.^1^ Response to antipsychotic therapy is highly variable, and it is not possible to predict those patients who will or will not respond to medications. Furthermore, around 30% of these patients are treatment-resistant.^2,4^ Across several studies, it has been found that the proportion of patients receiving antipsychotic polypharmacy ranged from 15.9–60.5% before they received clozapine (treatment-resistant schizophrenia).^3^ Treatment of these patients imposes a huge burden on the patient and the health system. Clozapine is the only approved and evidence-based drug treatment for treatment-resistant schizophrenia.

Antipsychotic drugs are the mainstay of acute and long-term schizophrenia treatment, but the treatment response and tolerability are highly variable.^11^ There are high rates of treatment discontinuation because of efficacy and tolerability issues.^12^ First-generation antipsychotics are often accompanied by significant adverse effects, including extrapyramidal symptoms and tardive dyskinesia. Second-generation antipsychotics are associated with various metabolic adverse effects, such as dyslipidemia, elevated glucose levels, and weight gain, and may cause clinical exacerbation or psychotic relapse, often resulting in hospital stays and placing a great burden on patients and their families. Antipsychotics represent a considerable fraction of healthcare costs in most developed countries.^13,14^ Pharmacogenomic (PGx) factors play a major role in deciding treatment responses to antipsychotic medicines.^2^ In past decades, research has succeeded in identifying genetic variants associated with variability in antipsychotic treatment.^15-18^

The presence of polymorphic alleles for cytochrome P (CYP) 450 may result in a lack of expression, altered levels of expression, or altered function of CYP450 enzymes. CYP2D6, CYP1A2, and CYP3A4/5 are major enzymes in the metabolism of antipsychotics, and polymorphisms of alleles for these proteins are associated with altered plasma levels. Consequently, standard dosing may result in drug plasma concentrations that are subtherapeutic or toxic in some patients. Patient CYP450 genotype testing can predict altered pharmacokinetics and is currently available and relatively inexpensive. Numerous studies have shown a significant association between genotype and adverse effects, such as CYP2D6 polymorphisms and tardive dyskinesia.^18^ Collectively, the literature provides a consistent body of evidence supporting the use of genotypic testing to prevent adverse events in adults receiving some antipsychotics. The role of additional genetic variants beyond CYP450 in the therapeutic and adverse responses to antipsychotics is currently being evaluated, including those polymorphisms related to pharmacodynamic targets such as dopamine and serotonin receptors, and cellular transporters.^19^ These studies offer additional avenues to predict efficacy and may be useful to prevent adverse effects, including weight gain and extrapyramidal symptoms. There are several genetic mechanisms for CYP450 metabolic phenotypes and their pharmacokinetic implications. Interestingly, the vast majority of common variants associated with treatment response and adverse effects of antipsychotics were identified only in the samples of European ancestry. The associated variants identified in populations of European ancestry might not be significant in other ancestry groups, because of underlying genetic heterogeneity.

The “trial-and error” method of selecting psychotropic agents for psychiatric patients implores the need to find ways that improve the current prescribing patterns for patients. Numerous genetic variants have been shown to be associated with antipsychotic response and adverse effects of schizophrenia treatment. The anticipation is that pharmacogenetic testing can be performed in patients with schizophrenia in the near future, prior to treatment initiation, in order to guide medication selection and prevent potential adverse effects. We have summarized the relevant literature in the global and Indian context in Tables 1-3.

Globally, the Clinical Pharmacogenetics Implementation Consortium guidelines are good tools by which to recommend the appropriate selection and dosing of a variety of medications, with those pertaining to psychiatry the most prominent. Different levels of metabolism are assigned to different gene variants (e.g., intermediate metabolizer, poor metabolizer, etc.). The development of activity scores can provide a better classification of different grades of metabolism with complexity; whereas medications are metabolized by more than one enzyme. Notably, the activity score may depend on the medication itself (substrate dependency).

Considering the Indian context, ethnic differences in pharmacogenomic variants have been well documented in literature and could significantly impact variability in response and adverse events to antipsychotic therapeutics. India is a large country with diverse ethnic populations of distinct genetic architecture. India’s national genome sequencing initiative (IndiGen) provides a unique opportunity to explore the landscape of pharmacogenomic variants using population‐scale whole genome sequences. Significant differences in the allele frequencies of clinically actionable pharmacogenomic variants in Indians were demonstrated when compared to the global populations. It was estimated, an average, each Indian individual carried eight pharmacogenomic variants (single nucleotide variants) that have a direct impact on the choice of treatment or drug dosing, including antipsychotic medicines. Clinically actionable pharmacogenomic variants and genes for which preemptive genotyping is most recommended for the Indian population has been also reported. Numerous genetic variants have been shown to be associated with antipsychotic response and adverse effects of schizophrenia treatment.^20^ Thus, overall, several gene variants related to antipsychotic response and adverse effects in the treatment of patients with schizophrenia have been reported, and several commercial pharmacogenomic tests have become available. However, further well-designed investigations and replication studies in large and well-characterized samples are needed to facilitate the application of pharmacogenomic findings to clinical practice.

**^
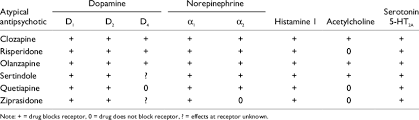
^**

**Fig. 1.** Primary receptors for actions of antipsychotics.^2^

#
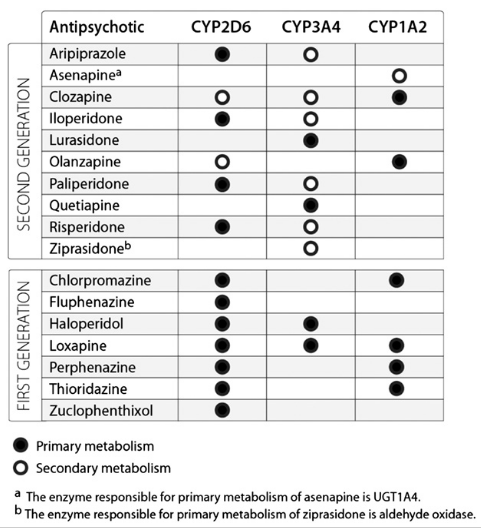


# Fig. 2. Primary and secondary CYP450 enzymes responsible for the metabolism of antipsychotics.^2^

#
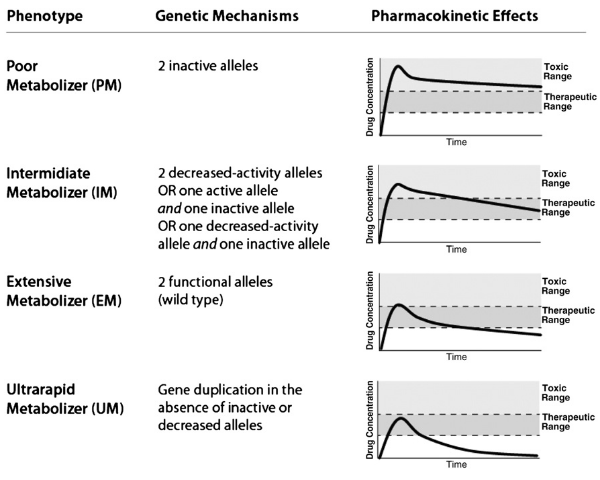


# Fig. 3. Genetic mechanisms for CYP450 metabolic phenotypes and their pharmacokinetic implication.^2^

Given the central role of the dopaminergic and serotonergic neurotransmitter systems in antipsychotic efficacy, the genes of these systems have received the greatest attention. The strongest support has accumulated for variation in genes encoding the dopamine D2 receptor (DRD2) dopamine D3 receptor (DRD3) serotonin 1A receptor (HTRIA) and serotonin 2A receptor (HTR2A). While a number of antipsychotics also show some affinity for receptors of the adrenergic, muscarinic, and histaminic systems, results from pharmacogenetic studies of these systems lack independent replication or are inconsistent. Outside of the classic neurotransmitter systems, zinc-finger domain-containing protein (ZNF)804A gained attention as a potential pharmacogenetic candidate following its identification as a risk locus in a schizophrenia genome-wide association study (GWAS). The disease-associated A allele of the ZNF804A rsl344706 polymorphisms was associated with abnormalities in brain connectivity among patients with schizophrenia. The precise biological functions of ZNF804A underlying its association with brain connectivity remain an active area of research. With respect to pharmacogenetics, an initial study reported no association between rsl344706 and overall antipsychotic response. However, two more recent studies have reported a significant association between the A allele and less improvement in positive symptoms. The association between rsl344706 and antipsychotic efficacy in the latter studies may be the direct result of an effect of ZNF804A on antipsychotic response, or an indirect result of this variant acting as a biomarker for more severe forms of schizophrenia presenting with greater treatment resistance. The effect sizes of genetic variants associated with antipsychotic response, considered as a binary outcome, have generally been modest (odds ratio, 0.18-0.82), such that none will predict antipsychotic response in a clinically meaningful way on their own. Such modest effect sizes are not surprising, given the complexity and polygenicity of drug response. The success of future efforts to improve the prediction of antipsychotic efficacy using genetic information will require the development of algorithms that incorporate multiple genetic factors, and their application in deeply phenotyped samples that can tease apart the heterogeneity in drug response as an outcome measure.^22^

About 75% of patients with schizophrenia discontinue their antipsychotic drug treatment within 18 months.^23^ Weight gain is a common and serious adverse effect of antipsychotic treatment. Around 30% of patients gain ≥7% of their baseline weight following antipsychotic treatment. There is robust evidence that variation in the HTR2C gene and melanocortin 4 (MC4R) receptors are associated with antipsychotic-induced weight gain, with moderate-to-large effect sizes. Tardive dyskinesia, a motor system disorder characterized by repetitive and involuntary movements, is a potentially irreversible adverse effect experienced by an estimated 25% of patients treated long-term with first-generation antipsychotics. There is evidence for a modest effect of CYP2D6, DRD2, and HTR2A on susceptibility to tardive dyskinesia. First identified in a GWAS, the association between variants in *heparan sulfate proteoglycan 2, perlecan* (HSPG2), and tardive dyskinesia was replicated in two independent samples. These initial results for HSPG2 highlight the potential utility of applying GWAS in well-phenotyped samples to identify novel candidate genes, which can then be followed up in subsequent replication studies.^22^

The efficacy of PGx-guided treatment in patients with depression has been demonstrated in several randomized controlled trials and meta-analyses. The findings indicate that treatment guided by PGx testing, including combinatorial testing, is superior to treatment as usual in response and/or remission rates in the acute treatment of depression.^24-31^ To date, no randomized controlled study (RCT) has been conducted to evaluate the outcomes in patients taking antipsychotics following the use of pharmacogenomic (PGx) guidance of treatment selections.

# Study Objectives

- To evaluate the clinical utility (safety and efficacy) of PGx-assisted treatment as compared to standard of care in terms of reduction in the incidence of solicited AEs and SAEs, requirement and duration of hospitalization, and time to respond in patients with schizophrenia.
- To estimate the cost-effectiveness of PGx-assisted treatment as compared to the standard of care in patients with schizophrenia.
- To explore the facilitators and challenges in implementing PGx-assisted treatment for schizophrenia.

# Investigational Plan

**Part 1**

- **Study design:** Randomized, parallel-arm, patient and assessor-blinded study.
- **Setting:** Department of Psychiatry, AIIMS, Kalyani.
- **Study population:** The following are the eligibility criteria:
  - **Inclusion criteria:** Adult (≥18 years) patients of both gender attending the OPD of Psychiatry, AIIMS, Kalyani, diagnosed with schizophrenia (DSM V), and are adjudicated by the treating psychiatrist to be treated with one or more of the following first-line antipsychotic medicine: olanzapine, risperidone, haloperidol, amisulpride, quetiapine, aripiprazole, and trifluoperazine.^32,33^
  - **Exclusion criteria:** Patients who are needed to be hospitalized or provided electroconvulsive therapy (ECT) at presentation, patients for whom any other antipsychotic drug is required to be initiated, and those who are unwilling to provide informed consent.
- **Treatment:** Variable-sized block randomization will be performed based on computer-generated random numbers. The recruited patients will be randomized (2: 1) into two arms:
  - **Group A:** PGx-assisted treatment (antipsychotic drug and dose selection depending on the results of the mentioned SNPs).
  - **Group B:** Standard of care (SOC).

The treating psychiatrists will be aware of the patient groups (unblinded). The patients will not be revealed their genotyping results (patient blind) until the study completion (follow-up for 12 weeks). Blood samples will be collected for all patients (Group A: PGx and levels of triglyceride, HDL cholesterol, and fasting blood sugar; Group B: levels of triglyceride, HDL cholesterol, and fasting blood sugar). The psychiatrists will be trained on the interpretation of the PGx test results prior to initiating the study. Until the genotyping results are available (expected in seven days), the patients will be initiated with the SOC. Antipsychotic selection and dose adjustment based n PGx information will be based on package inserts of US FDA, EMA, Health Canada, Pharmaceuticals and Medical Devices Agency, Japan; and professional PGx guidelines (e.g., Clinical Pharmacogenetics Implementation Consortium, Pharmacogenomics Knowledgebase, and Royal Dutch Association for the Advancement of Pharmacy – Pharmacogenetics Working Group), and primary scientific literature.^8,34,35^ The antipsychotic drug and dose in both groups may be altered based on the clinical response (efficacy or adverse events) as adjudicated by the treating psychiatrists during follow-up visits. The patients will be followed up for the next 12 weeks (at the end of weeks 2, 4, 8, and 12). The genotyping data will be revealed to the patients after the end of 12 weeks. If PGx-assisted treatment is beneficial, the same will be used for the SOC arm.

- **Genotyping:** After confirming eligibility, the patients will be recruited for the study. 3 ml of peripheral venous blood will be collected from patients of Group A. The following SNPs^34^ will be detected based on their role in the pharmacokinetics and pharmacodynamics of the mentioned antipsychotics and their prevalence in the eastern Indian population:

**Table 4.** Genes and respective SNPs that will be detected by genotyping.

| **Gene** | **SNP** | **Gene** | **SNP** |
| --- | --- | --- | --- |
| ***ABCB1*** | rs1045642 | ***CYP2D6*** | rs28371725 (CYP2D6*41) |
| ***ABCB1*** | rs2032582 | ***CYP2D6*** | rs3892097 (CYP2D6*4) |
| ***ABCB1*** | rs3842 | ***CYP3A5*** | rs776746 |
| ***CYP1A2*** | rs762551 | ***DRD2*** | rs1079597 |
| ***CYP2D6*** | rs1058164 | ***DRD2*** | rs2514218 |
| ***CYP2D6*** | rs1065852 (CYP2D6*10) | ***DRD2*** | rs4436578 |
| ***CYP2D6*** | rs1135840 (CYP2D6*10) | ***DRD2*** | rs6277 |
| ***CYP2D6*** | rs16947 (CYP2D6*14) | ***HTR1A*** | rs10042486 |
| ***CYP2D6*** | rs28371699 | ***HTR1A*** | rs6313 |
| ***CYP2D6*** | rs28371702 | ***HTR2C*** | rs1283677 |

Real-time quantitative Polymerase Chain Reaction (PCR) and Sanger sequencing will be used.^35^ Genomic DNA will be extracted from peripheral blood by using DNA Blood Mini Kit as per the instructions of the manufacturer. The respective gene loci will be amplified by using specific forward and reverse primers. The primer sequences will be checked for dimer and helix formation by using the Gene Runner software. PCR will be carried from 100 ng genomic DNA in a total reaction volume of 20 μl. The reaction mixture will consist of 2 μl of 10× buffer, 0.3 μl of 10 mM dNTP, 0.3 μl of each 10 mM forward and reverse primer, and 0.2 μl of 5U/μl of Taq polymerase. Nuclease-free water will be added to make the final reaction volume to 20 μl. The amplified products will be checked on 1.5% agarose gel electrophoresis. The polymorphisms will be classified as wild type, homozygous, or heterozygous mutation for the analysis.^35^

- **PGx-assisted antipsychotic medication selection and dosing algorithm:** Patients getting SOC (Group B) will be monitored as per standard practice. Based on the genotype results, the patients of Group A will be classified as:
  - **Poor metabolizer:** Two inactive alleles
  - **Intermediate metabolizer:** Two decreased activity alleles or one active and one inactive allele or one decreased activity and one inactive allele
  - **Fast metabolizer:** Two functional alleles (wild type)

Antipsychotic medication selection and dosing algorithm will be as follows:

- - ***ABCB1* polymorphism:** risperidone will be avoided
  - ***DRD2* polymorphism:** haloperidol, amisulpride will be avoided
  - ***HTR1A* polymorphism:** haloperidol, trifluoperazine will be avoided
  - ***HTR2C* polymorphism:** haloperidol, trifluoperazine, olanzapine will be avoided

The algorithm will be updated during the study, if required, based on the findings of interim analysis.

**Table 5.** PGx-assisted antipsychotic dosing.^4,8,34,35^ (Maudsley Guidelines).

| **Antipsychotic** | **Dose range (mg/day)^*^** |
| --- | --- |
| Olanzapine | First episode: 5; Multiple episode: 7.5 |
| Risperidone | First episode: 2; Multiple episode: 4 |
| Haloperidol | First episode: 2; Multiple episode: 4 |
| Amisulpride | First episode: 300; Multiple episode: 400 |
| Quetiapine | First episode: 150; Multiple episode: 300 |
| Aripiprazole | First episode: 10; Multiple episode: 10 |
| Trifluoperazine | First episode: 10; Multiple episode: 15 |


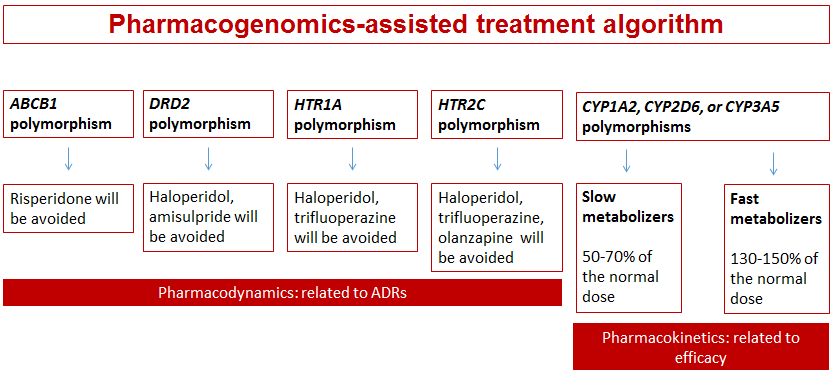


**Fig. 4.** PGx-assisted antipsychotic dosing.^4,8,9,34,35^. The pharmacokinetics-related dosage adjustment is based on the primary CYP450 metabolic enzymes as depicted in Fig. 2.

- **Withdrawal criteria:** If any patient is initiated treatment with any other antipsychotic drug apart from the drugs mentioned during the treatment or follow-up period or requires electroconvulsive therapy, the patient will be withdrawn from the study.
- **Therapeutic drug monitoring:** After reaching a steady state, TDM will be performed for each antipsychotic medicine using trough samples. This will be performed by the high throughput ultra-performance liquid chromatographic (UPLC) method. 100 mm X 2.1 mm id, 1.7 m particle size column will be used to separate the samples with gradient elution consisting of 0.1% tri-fluoro acetic acid and acetonitrile with a PDA detector. The elution time is expected to be 7 min with a 0.3 ml/min flow rate, 1 µl injection volume, and a 45°C column oven temperature.^36^ The Levey-Jennings chart will be used for QA/QC.
- **Assessment of parameters:** Outcome assessment will be performed by a psychiatrist/psychologist who will be unaware of the treatment groups of the patients. The schema of outcome assessment is represented in Table 6.
  - **At baseline:** Socio-demographic characteristics (gender, age, body mass index), addiction history (including smoking status), present medical history, present medication history, details of the present psychotic disorder (age of onset, duration, family history, severity of present illness as per PANSS and CGI, and treatment history).
  - **At 2, 4, 8, and 12 weeks:** Severity of illness (as per PANSS and CGI), the requirement of hospitalization, duration of hospitalization, serum drug levels, solicited AEs and SAEs, and medication adherence.
    - - **PANSS:** The Positive and Negative Syndrome Scale (PANSS) has been widely used in clinical trials of schizophrenia and other disorders and is considered the “gold standard” for the assessment of antipsychotic treatment efficacy. The PANSS has many items, evaluates a multidimensional array of symptoms (e.g. positive, negative, neuromotor, depressive), and involves the use of data from patient reports, caregiver reports, and clinical observations.^38^
      - **CGI:** The Clinical Global Impressions (CGI) Scale provides an overall clinician-determined summary measure that takes into account all available information, including knowledge of the patient's history, psychosocial circumstances, symptoms, behavior, and the impact of the symptoms on the patient's ability to function. The CGI comprises two companion one-item measures evaluating the following: severity of psychopathology from 1-7 and change from the initiation of treatment on a similar seven-point scale.^39^
      - **UKU-SERS:** The Udvalg for Kliniske Undersøgelser Side-Effect Rating Scale comprises ratings (0-4) of 48 single items, a global assessment of the influence of the reported adverse effects on daily performance, and a statement of the effects of the adverse events on the continuation of the medication. The items are clustered into four sub-groups: psychic, neurological, autonomic and, other adverse effects.^40^
      - **Solicited AEs and SAEs:** The following AEs will be collected: metabolic syndrome, extrapyramidal symptoms, sedation, sialorrhea, hyperprolactinemia, and sexual dysfunction.^41,42^ A 12-item SMARTS (Systematic Monitoring of Adverse events Related to TreatmentS) questionnaire will be also used.^43^ Metabolic syndrome will be evaluated by International Diabetes Federation (IDF) criteria at the end of 12 weeks. It includes the following: central obesity (defined as waist circumference with ethnicity-specific values) and any two of the following four factors (raised triglycerides, reduced HDL cholesterol, raised blood pressure, and raised fasting plasma glucose).^44^ Serum prolactin level will be also estimated.
      - **Medication adherence:** This will be assessed by the pill-counting method.^45^
      - **Treatment non-responsive:** <20% reduction in PANSS score at the end of 6 weeks as compared to the score at baseline.^46^
      - **EQ-5D-5L:** For the quality of life assessment, the descriptive system comprises five dimensions: mobility, self-care, usual activities, pain/discomfort, and anxiety/depression. Each dimension has 5 levels: no problems, slight problems, moderate problems, severe problems, and extreme problems.^47^
- **Outcomes:** Comparisons of the following parameters between the two groups through 12 weeks of follow-up:
  - Udvalg for Kliniske Undersøgelser Side-Effect Rating Scale (UKU-SERS) score
  - The proportion of patients developing solicited AEs and SAEs
  - The proportion of patients requiring hospitalization
  - Duration of hospitalization
  - Clinical response at week 12 (PANSS and CGI)
  - Time-to-achieve clinical response through 12 weeks (PANSS and CGI)
  - The proportion of patients non-responsive to treatment
  - Number and olanzapine-equivalent doses of various anti-psychotics
  - Medication adherence
  - Quality of life (EQ-5D-5L)
  - Correlation between serum drug level, dosing, and clinical response
- **Data Management:** The investigators will be responsible for assuring completeness, accuracy and timely collection of data. Case Record Forms (CRFs) should support the data entered in the electronic data capture system. They will be signed and dated by the person filling out the forms, and the investigator reviewing the same. Data will be reviewed by the investigator and co-signed. Data will be entered in REDCap software (Vanderbilt, USA) by the study sites and Case Record Forms will be scanned in REDCap.
- **Data Capture Methods:** Paper CRFs will be used to collect data. The data will then be entered on an electronic system. A data entry system with electronic tracking, password restricted access, audit trail, with time and date stamps on data entry and edits, will be developed. SOP for data collection will be provided. This SOP may be adopted by each site depending on their capacity and will have to be approved by ICMR.
- **Access to Source Data and Documents:** All data source documents, including clinical reports and records necessary for the evaluation and reconstruction of the clinical trial, will be stored securely, with a focus on ensuring the patient’s confidentiality.
- **Statistical analysis:** The data will be checked for normal distribution (Kolmogorov-Smirnov test). For categorical variables (e.g. response rates, safety outcomes) the chi-squared test will be used. For continuous variables (e.g. scale scores, duration of hospitalization), analysis of covariance (ANCOVA) with treatment in the model and baseline clinical and demographic characteristics and drug dosage as covariates will be used. Subgroup analysis will be performed based on the treatment-status (treatment naïve and who received prior treatment), educational level, negative symptoms from first psychotic episode, PANSS and CGI scores, comorbid substance use; age at onset, lack of early response, and adherence to treatment. The Kaplan-Meier estimate will be used to draw the survival curves denoting the time-to-achieve response. Patients will be labeled responsive if they have a ≥20% reduction in PANSS score at the end of 6 weeks as compared to the score at baseline.^46^ Cox's proportional hazards model will be used to assess the difference in the time to respond between the two groups allowing for other covariates. For correlation between drug dosing, drug levels, and clinical response Pearson’s or Spearman correlation test will be applied. A p-value of <0.05 will be considered significant. Interim analyses will be performed after the follow-up of 90, 180, and 270 patients. O'Brien-Fleming boundary will be used for stopping.

**Table 6.** Assessment of baseline and outcome parameters.

|  |  | **Screening** | **Enrolment (Baseline)** | **2 weeks** | **4 weeks** | **8 weeks** | **12 weeks** |
| --- | --- | --- | --- | --- | --- | --- | --- |
|  | **Screening and consent** | | | | | | |
|  | DSM V criteria for schizophrenia | x |  |  |  |  |  |
|  | **Post-enrolment** | | | | | | |
|  | Baseline demographic, clinical characteristics, and treatment history |  | x |  |  |  |  |
| **Objectives** | **Outcome parameters** | | | | | | |
| Solicited AEs and SAEs | UKU-SERS and SMARTS questionnaire |  |  |  | x | x | x |
|  | Metabolic syndrome (triglycerides, HDL cholesterol, blood pressure, and fasting plasma glucose levels) and serum prolactin level |  | x |  |  |  | x |
| Hospitalization | Frequency and duration |  |  | x | x | x | x |
| Clinical response | PANSS and CGI |  | x | x | x | x | x |
| Anti-psychotic medications | Olanzapine-equivalent doses |  |  | x | x | x | x |
| Medication adherence | Pill counting method |  |  |  | x |  | x |
| Quality of life | EQ-5D-5L |  | x |  |  |  | x |
| TDM | Serum drug level of each anti-psychotic |  |  | x |  |  |  |


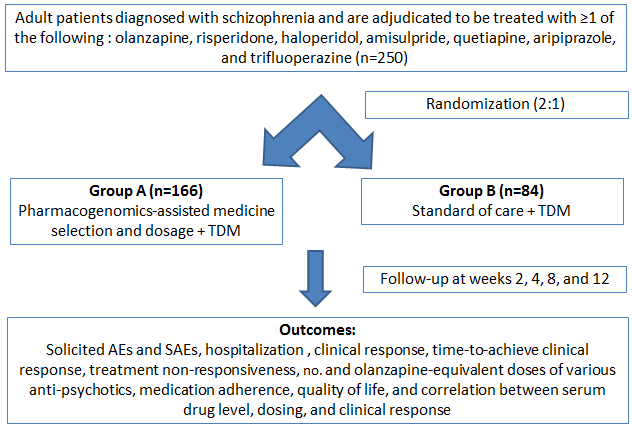


**Fig. 5.** Flowchart showing the study schema.

**Part 2**

- **Study design:** Cost-effectiveness analysis
- **Methodology and statistical analysis:** We will develop a decision analytic model from the health system’s perspective from the patients’ data using a short-term decision tree and a long-term (lifetime horizon) Markov model. The simulation will begin with a study of individuals entering the model as the target population and proceeding into one of two treatment strategies: PGx-assisted treatment and SOC. The Markov model structure will be developed as per the experience obtained from the trial as well as a literature review. The trial findings will be used as transitional probabilities for the decision tree and the Markov model. The costs of intervention will be estimated from the study with the use of a testing panel including respective gene-drug pairs with 30 test-return days. The other relevant model input parameters the utility values, the prevalence of schizophrenia, etc. will be retrieved from the literature and used in the models.

Table 7. The study PICOST.

| **Parameters** | **Details** |
| --- | --- |
| Population | Adult population diagnosed with schizophrenia (DSM V) |
| Intervention | PGx-assisted pharmacotherapy |
| Comparator | Standard treatment |
| Outcome | Incremental cost-utility ratio (ICUR) or incremental net benefit (INB) |
| Study type | Economic model-based cost-utility analysis (CUA) |
| Analytical time horizon | Lifetime horizon with a cycle length of one year |
| Perspective | Public Payer (government/healthcare provider) |
| Discounting | All future costs and consequences will be discounted at 3% per annum, along with sensitivity analysis with 0 to 6% per annum |

- The model will estimate the health outcomes in terms of life years, QALYs, and costs. We will use a lifetime horizon and a health system perspective. All future costs and consequences will be discounted at 3%. The costs will be reported in 2023 Indian rupees and also in US dollars. The effectiveness will be measured in quality-adjusted life years (QALYs). The outcome will be the incremental cost-effectiveness ratio (ICER = ΔCost/ΔQALY). One-way, scenario, and probabilistic sensitivity analyses will be performed to assess the robustness of the findings due to variations in demographics, risk levels, and follow-up timeframe. The intervention will be considered cost-effective if the ICER is lower than the willingness to pay threshold, fixed based on the per capita gross domestic product (GDP) as suggested by the WHO. A budget impact analysis will also be performed if the intervention is found to be cost-effective.


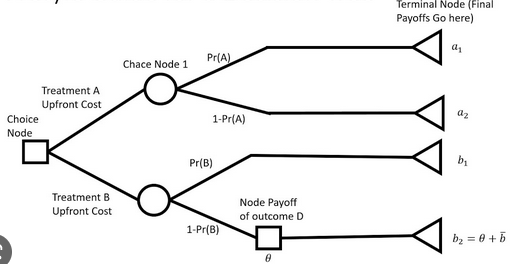


**Fig. 5.** Tentative decision tree model for cost-effectiveness analysis.

- **Budget impact analysis**: We will conduct a budget impact analysis of the implementation of the PGx intervention strategy only if it is found to be cost-effective (as mentioned previously). Budget impact analysis (BIA) will be conducted using the standard methods as per guidelines suggested by Health Technology Assessment India (HTAIn), Department of Health and Family Welfare, Government of India. We will estimate the annual budget impact for a period of 5 years. The input costs for the BIA will be taken from the previously estimated model (the decision-analytic Markov model). We will estimate the budget impact at the state level for each of the states of India considering the prevalence of Schizophrenia and state-level eligible patient population would be utilizing the PGx-assisted pharmacotherapy services assumed to be implemented at districts level hospitals. The budget required for offering the PGx-assisted pharmacotherapy services under the national program will be estimated using the following formula:

B=N*(Cdt+C_My1)

where,

B = Budget required for offering psychiatric treatment services to the eligible Population

N = Eligible population estimated using a top-down approach

Cdt = Unit cost of antipsychotic intervention from decision tree Markov model (dt)

CMy1 = Cost of schizophrenia management in first year from Markov (M)

- The estimated budget as the percentage of increase to be made from the existing total healthcare budget for each of the respective states would be provided.
- No discount will be applied as the budget impact is the estimation of the financial cost. The health budget will be projected based on a 5% annual increase in health expenditure, and the estimated budget for schizophrenia will be projected using the population’s annual growth rate until the year 2027. Further, the same method will be used to estimate the state-specific budget impact for the country-wide expansion of the treatment of patients with schizophrenia.

**Part 3**

- **Study design:** Qualitative study to explore the facilitators and challenges in implementing PGx-assisted treatment in patients with schizophrenia.
- **Methodology:** A narrative approach will be adopted. In-depth interviews (IDI) and focused-group discussions (FGD) of patients and their primary caregivers will be conducted. Key informant interviews (KII) will be conducted among psychiatrists. The interview guide will be prepared based on the following framework. A thematic analysis will be performed.^48-52^
  - General issues (facilitators and barriers) in the treatment of schizophrenia from the patient and familial perspectives
  - Impact on individual, family, and societal well-being
  - Medication adherence and ADRs
  - Hospitalization and the adverse consequences
  - The financial impact of treatment of schizophrenia
  - Expectations on precision medicine using PGx
  - Consideration of PGx-assisted treatment
  - Satisfaction with the quality of PGx testing and reports
  - Acceptability, feasibility, availability, and affordability of PGx-assisted treatment
  - Sustainability and scalability of PGx-assisted treatment
  - Facilitators and challenges in implementing PGx-assisted treatment

1. **Sample Size:**

Considering a difference of 0.62^11^ in the Udvalg for Kliniske Undersøgelser Side-Effect Rating Scale (UKU-SERS) score between the two groups, a standard deviation of 1.34^11^, alpha error of5%, power of 90%, a randomization ratio of 2: 1, and a drop-out rate of 10%, the final sample size is 250 (166 in PGx arm and 84 in SOC arm). This will give an opportunity for more patients to be enrolled in the PGx arm and will also allow detecting more SNPs. The following formula was used:


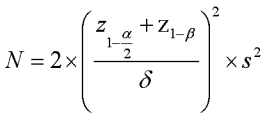


N=size per group; z_x_= the standard normal deviate for a one or two sided x; d= the real difference between two treatment effect; δ_0_= a clinically acceptable margin; S^2^= Polled standard deviation of both comparison groups.

For the qualitative study, we will interview 6-12 patients, 6-12 primary caregivers, and 6-12 treating physicians or till saturation is obtained.

1. **Project Implementation Plan**

- The patients will be recruited from the outpatient Department of Psychiatry, AIIMS, Kalyani. After the eligibility assessment, informed consent will be obtained. 3 ml of peripheral venous blood will be collected from the patients. The blood samples will be transferred to the Dept. of Biochemistry, AIIMS, Kalyani.
- In the Department of Biochemistry, AIIMS, Kalyani, the blood samples will be stored at -20^o^C until DNA extraction. After DNA extraction, genotyping will be performed using PCR and Sanger sequencing within 5 days. The genotyping results will be communicated to the treating Psychiatrist, based on which antipsychotic drug selection and dosing will be decided.
- After reaching a steady state, TDM will be performed using trough samples. After separating serum from the whole blood samples, the serum samples will be transported in dry ice on the same day to ICMR-Centre for Ageing and Mental Health, Kolkata. Serum drug levels (therapeutic drug monitoring) will be estimated at ICMR-Centre for Ageing and Mental Health, Kolkata using UPLC and the result will be communicated to the treating Psychiatrist. The Levey-Jennings chart will be used for QA/QC.
- The patients will be followed up for 12 weeks in the Department of Psychiatry, AIIMS, Kalyani for the pre-specified outcomes.
- After completing part I of the study, the cost-effectiveness analysis will be conducted at ICMR-National Institute of Epidemiology, Chennai, and ICMR-Centre for Ageing and Mental Health, Kolkata. ICMR-National Institute of Epidemiology, Chennai has a trained expert team on Health Technology Assessment.
- The qualitative study (IDI, KII, and FGD) will be conducted among patients, primary caregivers, and treating physicians in the Department of Psychiatry, AIIMS, Kalyani.

1. **Ethics**

The study will be initiated after obtaining IEC approval from ICMR-Centre for Ageing and Mental Health, Kolkata, and AIIMS, Kalyani. All participants will be recruited after obtaining written informed consent. The study will conform to the requirements of the Declaration of Helsinki, 1964; Indian GCP; and ICMR-National Ethical Guidelines for Biomedical and Health Research Involving Human Participants (2017). The genotyping data will be revealed to the patients after 12 weeks. If PGx-assisted treatment is beneficial, it will be used for the SOC arm. A 2: 1 randomization technique will be used. This will give an opportunity for more patients to be enrolled in the PGx arm and get benefitted if PGx-assisted treatment is beneficial.

1. **Expected Outcomes (up to 100 words)**

It is important to ensure effective and safe treatment for patients with schizophrenia. Treatment optimization by using the concept of precision medicine is required to improve the efficacy and reduce the development of ADRs in patients on antipsychotic therapy. The findings of this study will help in understanding whether PGx-assisted treatment (drug and dosage selection) supported by therapeutic drug monitoring has a clinical utility (safety and efficacy) or is cost-effective in patients with schizophrenia. This study is a hybrid type 2 effectiveness-implementation research. If the results are positive, PGx-assisted treatment might be implemented in the Standard Treatment Guideline for the treatment of- schizophrenia.

1. **Limitations of this study (up to 100 words)**

- The variability of antipsychotic response cannot be fully explained by PGx. Instead of GWAS, a candidate gene approach will be taken considering the most relevant SNPs (associated with the pharmacokinetics and pharmacodynamics of the mentioned antipsychotics) which are prevalent in the eastern Indian population.
- Ensuring the generalizability of the findings across India, including the diverse Indian population (having different genotypes) could not be performed because of serious logistic challenges. The genotyping will be performed in a single laboratory, and based on the genotyping result, PGx-assisted treatment will be promptly initiated, avoiding delay in the treatment.
- The exact intervention (PGx-assisted treatment) is still empirical (trial and error) and has not been adjudicated by any international society or guidelines; hence, the development of optimal drug selection and dosing algorithm requires a large volume of data.
- PGx-assisted treatment optimization is an evolving discipline. Relevant expertise and large-scale implementation of PGx-assisted treatment requires manifold further research and development.

1. **Future plans based on expected outcomes if any (up to 100 words)**

- If PGx-assisted treatment has a clinical utility in terms of effectiveness and safety, the study will be extended to the pan-Indian population having diverse genotypes to assess the generalizability of the findings.
- Further, the PGx-assisted treatment algorithm will be developed more robustly as more data emerge from the study. An approach of precision medicine will be taken by developing a robust model to guide antipsychotic selection and dosing considering all variables in patients with schizophrenia.
- Efforts will be made to address the challenges in implementing PGx-assisted antipsychotic selection dosing on a large scale and make it cost-effective.

1. **Timelines**

| **Activities** | **Year 1** | | | | **Year 2** | | | | **Year 3** | | | |
| --- | --- | --- | --- | --- | --- | --- | --- | --- | --- | --- | --- | --- |
|  | **Q1** | **Q2** | **Q3** | **Q4** | **Q1** | **Q2** | **Q3** | **Q4** | **Q1** | **Q2** | **Q3** | **Q4** |
| Approvals and formalities |  |  |  |  |  |  |  |  |  |  |  |  |
| Recruitment of staff and training |  |  |  |  |  |  |  |  |  |  |  |  |
| Purchase of items |  |  |  |  |  |  |  |  |  |  |  |  |
| Data collection: part 1 |  |  |  |  |  |  |  |  |  |  |  |  |
| Data analysis: part 1 |  |  |  |  |  |  |  |  |  |  |  |  |
| Conduct and analysis: part 2 |  |  |  |  |  |  |  |  |  |  |  |  |
| Conduct and analysis: part 3 |  |  |  |  |  |  |  |  |  |  |  |  |
| Report and result dissemination |  |  |  |  |  |  |  |  |  |  |  |  |

1. **Institutional Support**

- The patients will be recruited from the Department of Psychiatry, AIIMS, Kalyani. There is a footfall of around 50 patients with schizophrenia in the OPD per month. The Department of Psychiatry, AIIMS, Kalyani has an indoor facility of 20 beds. So, in two years, the required sample size (n=250) is expected to be achieved.
- The blood samples from the patients will be collected from the Department of Psychiatry, AIIMS, Kalyani and transferred to the Department of Biochemistry, AIIMS, Kalyani for genotyping. The Department of Biochemistry, AIIMS, Kalyani has a running facility for storing blood samples, extracting DNA, and performing genotyping using PCR and Sanger sequencing. The genotyping results will be communicated to the treating Psychiatrist within 5 days, based on which antipsychotic drug selection and dosing will be decided.
- Serum drug levels (therapeutic drug monitoring) will be estimated at ICMR-Centre for Ageing and Mental Health, Kolkata. The Centre has a well-established laboratory and trained scientists and technicians to perform therapeutic drug monitoring.
- The cost-effectiveness analysis will be conducted at ICMR-National Institute of Epidemiology, Chennai and ICMR-Centre for Ageing and Mental Health, Kolkata. ICMR-National Institute of Epidemiology, Chennai has a trained expert team on Health Technology Assessment.
- The qualitative study (IDI, KII, and FGD) will be conducted among patients, primary caregivers, and treating physicians in the Department of Psychiatry, AIIMS, Kalyani.

# References

1. National Mental Health Survey of India, 2015-16. Ministry of Health and Family Welfare. Available at: <http://indianmhs.nimhans.ac.in/Docs/Report2.pdf> (accessed on 17.02.2023).
2. Yoshida K, Müller DJ. Pharmacogenetics of Antipsychotic Drug Treatment: Update and Clinical Implications. Mol Neuropsychiatry. 2020;5(Suppl 1):1-26.
3. Patel MX, Bishara D, Jayakumar S, et al. Quality of prescribing for schizophrenia: evidence from a national audit in England and Wales. Eur Neuropsychopharmacol. 2014;24:499-509.
4. Beunk L, et al. Dutch Pharmacogenetics Working Group (DPWG) guideline for the gene-drug interaction between CYP2D6, CYP3A4 and CYP1A2 and antipsychotics. Eur J Hum Genet. 2023 Mar 31. doi: 10.1038/s41431-023-01347-3.
5. FDA: Table of Pharmacogenomic Biomarkers in Drug Labeling. Available at: <https://www.fda.gov/drugs/science-and-research-drugs/table-pharmacogenomic-biomarkers-drug-labeling> (accessed on 17.02.2023).
6. PharmGKB. <https://www.pharmgkb.org> (accessed on 17.02.2023)
7. DPWG: Dosing Guidelines. Available at: <https://www.pharmgkb.org/guidelineAnnotations> (accessed on 17.02.2023)
8. Jürgens G, Andersen SE, Rasmussen HB, et al. Effect of Routine Cytochrome P450 2D6 and 2C19 Genotyping on Antipsychotic Drug Persistence in Patients With Schizophrenia: A Randomized Clinical Trial. JAMA Netw Open. 2020 Dec 1;3(12):e2027909.
9. Arranz MJ, Gonzalez-Rodriguez A, Perez-Blanco J, et al. A pharmacogenetic intervention for the improvement of the safety profile of antipsychotic treatments. Transl Psychiatry. 2019;9:177.
10. Karamperis K, Koromina M, Papantoniou P, et al. Economic evaluation in psychiatric pharmacogenomics: a systematic review. Pharmacogenomics J. 2021;21:533-541.
11. Huber CG, Naber D, Lambert M. Incomplete remission and treatment resistance in first-episode psychosis: definition, prevalence and predictors. Expert Opin Pharmacother 2008; 9:2027-38.
12. Lieberman JA. Effectiveness of antipsychotic drugs in patients with chronic schizophrenia: efficacy, safety and cost outcomes of CATIE and other trials. J Clin Psychiatry 2007; 68:e04.
13. Jones PB, Barnes TR, Davies L, et al. Randomized controlled trial of the effect on quality of life of second- vs first-generation antipsychotic drugs in schizophrenia: cost utility of the latest antipsychotic drugs in schizophrenia study (CUtLASS 1). Arch Gen Psychiatry 2006; 63:1079-87.
14. Ilyas S, Moncrieff J. Trends in prescriptions and costs of drugs for mental disorders in England, 1998-2010. Br J Psychiatry 2012; 200:393-8.
15. Lencz T, Robinson DG, Xu K, et al. DRD2 promoter region variation as a predictor of sustained response to antipsychotic medication in first-episode schizophrenia patients. Am J Psychiatry 2006; 163(3):529-31.
16. Zhang J-P, Lencz T, Malhotra AK. D2 receptor genetic variation and clinical response to antipsychotic drug treatment: a meta-analysis. Am J Psychiatry 2010; 167:63-72.
17. Reynolds GP, Templeman LA, Zhang ZJ. The role of 5-HT2C receptor polymorphisms in the pharmacogenetics of antipsychotic drug treatment. Prog Neuro Psychopharmacol Biol Psychiatry 2005; 29:1021-8
18. Ravyn D, Ravyn V, Lowney R, Nasrallah HA. CYP450 pharmacogenetic treatment strategies for antipsychotics: a review of the evidence. Schizophr Res 2013; 149:1-14
19. Arranz MJ, Rivera M, Munro JC. Pharmacogenetics of response to antipsychotics in patients with schizophrenia. CNS Drugs. 2011;25:933-969.
20. Sahana S, Bhoyar RC, Sivadas A, et al. Pharmacogenomic landscape of Indian population using whole genomes. Clin Transl Sci. 2022;15:866-877.
21. van der Weide J, van Baalen-Benedek EH, Kootstra-Ros JE. Metabolic ratios of psychotropics as indication of cytochrome P450 2D6/2C19 genotype. Ther Drug Monit. 2005;27:478-483.
22. Pouget JG, Shams TA, Tiwari AK, Müller DJ. Pharmacogenetics and outcome with antipsychotic drugs. Dialogues Clin Neurosci. 2014;16:555-66.
23. Lieberman JA, Stroup TS, McEvoy JP, et al. Effectiveness of antipsychotic drugs in patients with chronic schizophrenia. N Engl J Med. 2005;353:1209-1223.
24. Greden JF, Parikh SV, Rothschild AJ, et al. Impact of pharmacogenomics on clinical outcomes in major depressive disorder in the GUIDED trial: A large, patient- and rater-blinded, randomized, controlled study. J Psychiatr Res. 2019;111:59-67.
25. Perlis RH, Dowd D, Fava M, et al. Randomized, controlled, participant- and rater-blind trial of pharmacogenomic test-guided treatment versus treatment as usual for major depressive disorder. Depress Anxiety. 2020;37:834-841.
26. Vande Voort JL, Orth SS, Shekunov J, et al. A Randomized Controlled Trial of Combinatorial Pharmacogenetics Testing in Adolescent Depression. J Am Acad Child Adolesc Psychiatry. 2022;61:46-55.
27. Pérez V, Salavert A, Espadaler J, et al.; AB-GEN Collaborative Group; Menchón JM. Efficacy of prospective pharmacogenetic testing in the treatment of major depressive disorder: results of a randomized, double-blind clinical trial. BMC Psychiatry. 2017;17:250.
28. Thase ME, Parikh SV, Rothschild AJ, et al. Impact of Pharmacogenomics on Clinical Outcomes for Patients Taking Medications With Gene-Drug Interactions in a Randomized Controlled Trial. J Clin Psychiatry. 2019;80:19m12910.
29. Tiwari AK, Zai CC, Altar CA, et al. Clinical utility of combinatorial pharmacogenomic testing in depression: A Canadian patient- and rater-blinded, randomized, controlled trial. Transl Psychiatry. 2022;12:101.
30. Bradley P, Shiekh M, Mehra V, et al. Improved efficacy with targeted pharmacogenetic-guided treatment of patients with depression and anxiety: A randomized clinical trial demonstrating clinical utility. J Psychiatr Res. 2018;96:100-107.
31. Brown L, Vranjkovic O, Li J, et al. The clinical utility of combinatorial pharmacogenomic testing for patients with depression: a meta-analysis. Pharmacogenomics. 2020;21:559-569.
32. Grover S, Avasthi A. Anti-psychotic prescription pattern: A preliminary survey of Psychiatrists in India. Indian J Psychiatry. 2010;52:257-9.
33. Grover S, Avasthi A, Sinha V, et al. Indian Psychiatric Society multicentric study: Prescription patterns of psychotropics in India. Indian J Psychiatry. 2014;56:253-64.
34. Clinical Pharmacogenetics Implementation Consortium (CPIC®). Available from: <https://cpicpgx.org/genes-drugs/> (accessed on 24/03/2023).
35. Genomind Pharmacogenetic Report. Available from: <https://www.ncbi.nlm.nih.gov/gtr/tests/523653/methodology/> (accessed on 24/03/2023).
36. Jebaliya H, Shah A, Karad SC, et al. Quantification of panel of most potent antipsychotic medicines by high throughput UPLC method. Results Chem. 2022;4:100427.
37. Huhn M, Nikolakopoulou A, Schneider-Thoma J, et al. Comparative Efficacy and Tolerability of 32 Oral Antipsychotics for the Acute Treatment of Adults With Multi-Episode Schizophrenia: A Systematic Review and Network Meta-Analysis. Focus (Am Psychiatr Publ). 2020;18:443-455.
38. Kay SR, Fiszbein A, Opler LA. The Positive and Negative Syndrome Scale (PANSS) for schizophrenia. Schizophr Bull. 1987;13:261-276.
39. Guy W, editor. ECDEU Assessment Manual for Psychopharmacology. Rockville, MD: US Department of Health, Education, and Welfare Public Health Service Alcohol, Drug Abuse, and Mental Health Administration; 1976.
40. Lingjaerde O, Ahlfors UG, Bech P, et al. The UKU side effect rating scale. A new comprehensive rating scale for psychotropic drugs and a cross-sectional study of side effects in neuroleptic-treated patients. Acta Psychiatr Scand Suppl. 1987;334:1-100.
41. Muench J, Hamer AM. Adverse effects of antipsychotic medications. Am Fam Physician. 2010;81:617-22.
42. Stroup TS, Gray N. Management of common adverse effects of antipsychotic medications. World Psychiatry. 2018;17:341-356.
43. Haddad PM, Fleischhacker WW, Peuskens J, et al. SMARTS (Systematic Monitoring of Adverse events Related to TreatmentS): The development of a pragmatic patient-completed checklist to assess antipsychotic drug side effects. Ther Adv Psychopharmacol. 2014;4:15-21.
44. Alberti KG, Zimmet P, Shaw J. Metabolic syndrome--a new world-wide definition. A Consensus Statement from the International Diabetes Federation. Diabet Med. 2006;23:469-80.
45. Lee Y, Lee MS, Jeong HG, et al. Medication Adherence Using Electronic Monitoring in Severe Psychiatric Illness: 4 and 24 Weeks after Discharge. Clin Psychopharmacol Neurosci. 2019;17:288-296.
46. Kane JM, Agid O, Baldwin ML, et al. Clinical Guidance on the Identification and Management of Treatment-Resistant Schizophrenia. J Clin Psychiatry. 2019 Mar 5;80:18com12123.
47. EQ-5D. Available at: <https://euroqol.org/eq-5d-instruments/eq-5d-5l-about/> (accessed on 17.02.2023)
48. Cooper RE, Hanratty É, Morant N, et al. Mental health professionals' views and experiences of antipsychotic reduction and discontinuation. PLoS One. 2019;14:e0218711.
49. Lewins A, Morant N, Akther-Robertson J, et al. A qualitative exploration of family members' perspectives on reducing and discontinuing antipsychotic medication. J Ment Health. 2022:1-8.
50. Younas M, Bradley E, Holmes N, et al. Mental health pharmacists views on shared decision-making for antipsychotics in serious mental illness. Int J Clin Pharm. 2016;38:1191-9.
51. Crellin NE, Priebe S, Morant N, et al. An analysis of views about supported reduction or discontinuation of antipsychotic treatment among people with schizophrenia and other psychotic disorders. BMC Psychiatry. 2022;22:185.
52. Bondre AP, Shrivastava R, Raghuram H, et al. A qualitative exploration of perceived needs and barriers of individuals with schizophrenia, caregivers and clinicians in using mental health applications in Madhya Pradesh, India. SSM Ment Health. 2022;2:100063.

# Budget

**Justification of Budget**

| **Head** | **Details** | **Year 1** | **Year 2** | **Year 3** | **Amount (INR)** | **Justification** |
| --- | --- | --- | --- | --- | --- | --- |
| Staff/Manpower | Project TO (Psychologist) (**two**) (Rs 32,000/month + 10% annual increment) | 7,68,000 | 8,44,800 | - | 16,12,800 | For patient recruitment, consent, and overall coordination |
|  | Research Assistant (**two**) (Rs 31,000/month + 10% annual increment) | 7,44,000 | 8,18,400 | - | 15,62,400 | For genotyping and TDM sample preparation (laboratory works) |
|  | Research Assistant (one) (Rs 31,000/month | - | - | 3,72,000 | 3,72,000 | For cost-effectiveness analysis at ICMR-NIE, Chennai |
|  | Research Associate III (**one**) (Rs 68,580/month + 10% annual increment) | 8,22,960 | 9,05,256 | 9,96,144 | 27,24,360 | For overall study co-ordination and monitoring |

| **Head** | **Details** | | **Year 1** | | **Year 2** | | **Year 3** | | **Amount (INR)** | | **Justification** | |  |
| --- | --- | --- | --- | --- | --- | --- | --- | --- | --- | --- | --- | --- | --- |
| Equipment | Laptop (**one**) (Rs 1,00,000) | | 90,000 | | - | | - | | 90,000 | | The PI does not have a computer system provided by the office. One dedicated laptop is necessary. A non-availability certificate will be provided. The PI needs to travel to AIIMS, Kalyani from ICMR-CAM, Kolkata for continuous study co-ordination and monitoring. AIIMS, Kalyani is the site where all the patients will be recruited and treated. | |  |
|  | Printer (**one**) (Rs 25,000) | | 25,000 | | - | | - | | 25,000 | | For printing study documents | |  |
| **Head** | | **Details** | | **Year 1** | | **Year 2** | | **Year 3** | | **Amount (INR)** | | **Justification** | |
| Contingency | | Laboratory investigations | | 2,50,000 | | 2,50,000 | | - | | 5,00,000 | | For the estimation of triglycerides, HDL, FBS, and serum prolactin levels of all patients twice **(money to be transferred to AIIMS, Kalyani)** | |
|  |  | Sample transport cost | | 1,00,000 | | 1,00,000 | | 1,00,000 | | 3,00,000 | | For serum sample transport for TDM from AIIMS, Kalyani to ICMR-CAM, Kolkata | |
|  |  | Travel of PI from ICMR-CAM, Kolkata to AIIMS, Kalyani and ICMR-NIE, Chennai | | 2,00,000 | | 2,00,000 | | 2,00,000 | | 6,00,000 | | Travel of PI from ICMR-CAM, Kolkata to AIIMS, Kalyani and ICMR-NIE, Chennai for study co-ordination and monitoring | |
|  |  | Conducting meetings with experts | | 20,000 | | 20,000 | | 20,000 | | 60,000 | | For providing TA/DA to experts | |
|  |  | Printing and stationaries | | 20,000 | | 10,000 | | 10,000 | | 40,000 | | For printing study documents | |
|  |  | Publication-related expenses | | - | | 75,000 | | 75,000 | | 1,50,000 | | For publication-related expenses | |
|  |  | Other contingencies | | 20,000 | | 20,000 | | 10,000 | | 50,000 | | Compensation for patients, etc. | |

| **Head** | **Details** | **Year 1** | **Year 2** | **Year 3** | **Amount (INR)** | **Justification** |
| --- | --- | --- | --- | --- | --- | --- |
| Consumables | Consumables and plastic wares for PGx study | 10,00,000 | 10,00,000 | - | 20,00,000 | Consumables and plastic wares for genotyping **(money to be transferred to AIIMS, Kalyani)** |
|  | Consumables and plastic wares for sample preparation for TDM | 8,00,000 | - | - | 8,00,000 | Consumables and plastic wares for sample preparation for TDM |
|  | Estimation of drug levels (**outsourcing**) | 2,81,250 | 2,81,250 | - | 5,62,500 | Three anti-psychotic drug levels for 250 patients at INR 750/sample |

| **Head** | **Details** | **Year 1** | **Year 2** | **Year 3** | **Amount (INR)** |  |
| --- | --- | --- | --- | --- | --- | --- |
| **Grand Total** | | 51,41,210 | 45,24,706 | 17,83,144 | 1,14,49,060 |  |

**Appendix**

**1. Positive and Negative Syndrome Scale** (**PANSS**)

To assess a patient using PANSS, an approximately 45-minute clinical interview is conducted. The patient is rated from 1 to 7 on 30 different symptoms based on the interview as well as reports of family members or primary care hospital workers.

### Positive scale

7 Items, (minimum score = 7, maximum score = 49)

- Delusions
- Conceptual disorganization
- Hallucinations
- Excitement
- Grandiosity
- Suspiciousness/persecution
- Hostility

### Negative scale

7 Items, (minimum score = 7, maximum score = 49)

- Blunted affect
- Emotional withdrawal
- Poor rapport
- Passive/apathetic social withdrawal
- Difficulty in abstract thinking
- Lack of spontaneity and flow of conversation
- Stereotyped thinking

### General Psychopathology scale

16 Items, (minimum score = 16, maximum score = 112)

- Somatic concern
- Anxiety
- Guilt feelings
- Tension
- Mannerisms and posturing
- Depression
- Motor retardation
- Uncooperativeness
- Unusual thought content
- Disorientation
- Poor attention
- Lack of judgment and insight
- Disturbance of volition
- Poor impulse control
- Preoccupation
- Active social avoidance

PANSS Total score minimum = 30, maximum = 210


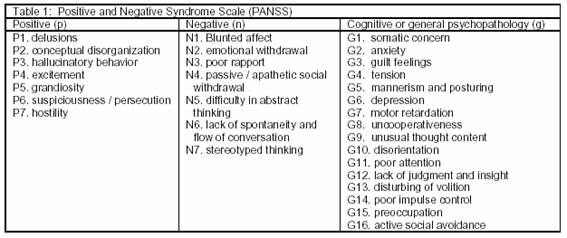


Kay SR, Fiszbein A, Opler LA. The Positive and Negative Syndrome Scale (PANSS) for schizophrenia. Schizophr Bull. 1987;13:261-276.

**2. Clinical global impression** (**CGI**) **scale**

**Severity scale**

- - Normal, not at all ill
  - Borderline mentally ill
  - Mildly ill
  - Moderately ill
  - Markedly ill
  - Severely ill
  - Among the most extremely ill patients

**Improvement scale**

- - Very much improved
  - Much improved
  - Minimally improved
  - No change
  - Minimally worse
  - Much worse
  - Very much worse

**Efficacy index**

**Therapeutic effect**

Marked – Vast improvement. Complete or nearly complete remission of all symptoms

Moderate – Decided improvement. Partial remission of symptoms

Minimal – Slight improvement which doesn't alter status of care of patient

Unchanged or worse

**Side effects**

None

Do not significantly interfere with patient's functioning

Significantly interfere with patient's functioning

Outweigh therapeutic effect

**
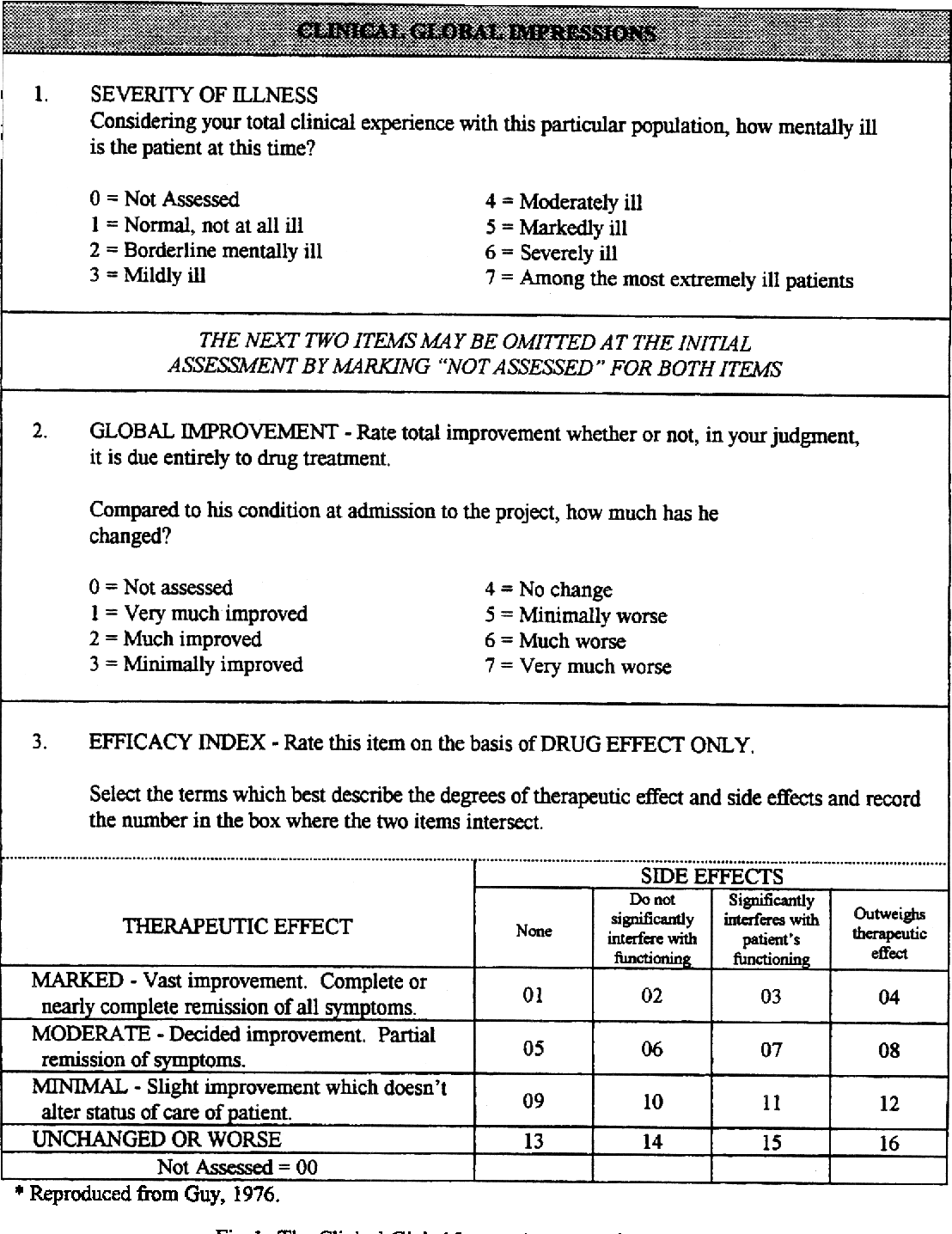
**

Guy W, editor. ECDEU Assessment Manual for Psychopharmacology. Rockville, MD: US Department of Health, Education, and Welfare Public Health Service Alcohol, Drug Abuse, and Mental Health Administration; 1976.

**UKU-SERS**

- - Psychic functions
    1.1 Do you have difficulties in collecting your thoughts, or understanding the context when you are reading, talking to somebody, watching TV or listening to the radio?
    Not at all
    A little more than usual
    More than usual
    Much more than usual
    1.2 Do you feel tired, or do you quickly become exhausted, or do you need to rest
    often in order to manage to continue your activities?
    Not at all
    A little more than usual
    More than usual
    Much more than usual
    1.3 Do you feel sleepier than usual, or is it difficult staying awake during the daytime?
    Not at all
    A little more than usual
    More than usual
    Much more than usual
    1.4 Have you noticed that you are more forgetful than usual, or that you do not
    remember simple things, or that your memory fails you?
    Not at all
    A little more than usual
    More than usual
    Much more than usual
    1.5 Do you have feelings of sadness, depression, listlessness/ dispiritedness or meaninglessness?
    Not at all
    Somewhat more than usual
    More than usual
    Much more than usual
    1.6 Do you feel nervous, restless, tense, or do you have trouble relaxing?
    Not at all
    A little more than usual
    More than usual
    Much more than usual
    1.7 Do you sleep more, longer or heavier than usual?
    Not at all
    Slightly more than usual
    More than usual
    Much more than usual
    1.8 Do you sleep less or less deeply than usual?
    Not at all
    A little more than usual
    More than usual
    Much more than usual
    1.9 Are you dreaming more often or more vividly than usual?
    Not at all
    A little more than usual
    More than usual
    Much more than usual
    1.10 Do you have a feeling of indifference or apathy for things happening around you?
    Not at all
    A little more than usual
    More than usual
    Much more than usual
  - Neurological signs/symptoms
    2.1 Are you troubled by muscle ache, tension or cramps anywhere in your body?
    Not at all
    Mild, occasionally
    Moderate, yes definitely
    Severe, frequently
    2.2 Are you troubled by stiffness/rigidity in your muscles when resting or moving?
    Not at all
    Mild, occasionally
    Moderate, yes definitively
    Severe, frequently
    2.3 Are you troubled by difficulty moving, or are your movements slower/more
    sluggish than usual?
    Not at all
    A little more than usual
    More than usual
    Much more than usual
    2.4 Are you troubled more often than usual by involuntary movements/spasms, for
    instance in your head and neck, face, arms, legs or upper body?
    No, not at all
    Yes, occasionally
    Yes, quite often
    Yes, almost constantly
    2.5 Are you troubled more often than usual by tremors or shakings in your hands, feet
    or elsewhere?
    No, not at all
    Yes, occasionally
    Yes, quite often
    Yes, almost constantly
    2.6 Have you noticed an increased urge to move about, to keep walking around, or
    difficulty sitting still or standing still?
    Not at all
    I like to keep moving around, but have no difficulty sitting or standing still
    I have to force myself to sit down or stand still
    I have to keep walking around all the time
    2.7 Have you experienced fainting spells or short blackouts, or have you had a seizure with loss of consciousness?
    Not at all
    Occasionally
    On several occasions
    Daily, one or more times each day
    2.8 Do you suffer from pricking, tingling or burning sensations in your skin anywhere
    on your body?
    Not at all
    Occasionally
    Yes, often
    Almost all the time
    2.9 Do you suffer headaches more often or more severely than usual?
    Not at all
    A little more than usual
    More than usual
    Much more than usual
  - Autonomous side effects
    3.1 Have you been troubled more than usual by difficulties in focussing (blurred vision), for instance when reading, writing by hand, knitting, embroidering, working with crochet or comparable?
    Not at all
    Some difficulty
    Can only read large letters, or work with larger objects
    Cannot read or do handicraft work at all
    3.2 Have you been troubled by increased salivation (mouth watering)?
    Not at all
    Increased salivation, but not a problem
    Must spit often
    Profuse salivation. I must often wipe my mouth. Pillow gets wet at night while
    sleeping.
    3.3 Has a dry mouth troubled you?
    Not at all

A little more than usual
More than usual
Much more than usual
3.4 Have you been troubled by nausea (feeling sick) and/or vomiting?
Not at all
Some nausea
Severe nausea
Vomited on one or more occasions
3.5 Has loose stools or diarrhoea troubled you?
Not at all
A little, but not problematic
Need to empty my bowels frequently
Severe diarrhoea, difficulty keeping continence
3.6 Have you been troubled by constipation?
Not at all
A little, but not problematic
Constipated
Constipated, need to take laxatives
3.7 Have you experienced difficulties in passing your urine?
Not at all
Difficulty in beginning urination
The flow is weak. It takes longer than usual to empty my bladder
Cannot empty my bladder, need help
3.8 Do you need to urinate more frequently than normally and drink water more often
than usual?
Not at all
Yes, must urinate more often, have to get up at night
Yes, have to urinate several times day and night, often thirsty
Yes, very often, even at night, need to drink frequently
3.9 Does dizziness or fainting fits when getting up from a lying or sitting position
trouble you?
Not at all
Sometimes, but I can stand up without problems
Must rise slowly from sitting or lying positions
Difficulty in standing up due to dizziness or feeling faint
3.10 Do palpitations or irregular heartbeats trouble you?
Not at all
Occasionally, not troublesome
Often, troublesome
Very often, severe problem
3.11 Does increased body sweat trouble you?
Not at all
A little more than normal
More than normal
Much more than normal

- - Other side effects
    4.1 Do you have, or have you had a rash?
    Not at all
    Light rash on limited area
    Rash on part of body
    Break out all over body
    4.2 Does itching trouble you?
    Not at all
    Light itching
    Severe itching
    Very severe, must scratch constantly
    4.3 Have you noticed any increased sensitivity to sunlight (reddening of the skin, severe sunburn)?
    Not at all
    A little more than usual
    More pronounced than usual, irritating
    So pronounced and irritating that my medication had to be withdrawn
    4.4 Have you noticed any skin discoloration (brown or other colour), localised to parts
    of skin exposed to light?
    Not at all
    Slight increase in pigmentation
    Marked increase in pigmentation

So pronounced pigmentation that other people have made remarks about it
4.5 Have you gained weight during the past four weeks?
Not at all
Gained 1-2 kg (2-4 pounds)
Gained 3-4 kg (6-8 pounds)
Gained more than 4 kg (more than 8 pounds)
4.6 Have you lost weight during the past month?
Not at all
Lost 1-2 kg (2-4 pounds)
Lost 3-4 kg (6-8 pounds)
Lost more than 4 kg (more than 8 pounds)
4.9 Have you noticed milk from your nipples?
Not at all
Some
Yes, but not troublesome
Much, stains my underwear
4.10 Have you experienced tension or swelling in your breasts?
Not at all
Some tension and swelling
Breasts are tense and larger than normal
Breasts clearly enlarged
4.11 Have you experienced increased sexual interest or increased sexual desire?
Not at all
Somewhat more than normal
More than normal
Much more than normal
4.12 Have you experienced decreased sexual interest or decreased sexual desire?
Not at all
A little less than normal
Less than normal
Much less than normal

- - Females only
    4.7a Have you noticed more discharge/bleeding when menstruating?
    Not at all
    Somewhat more than normally
    More than normally
    Profuse discharge/bleeding
    4.7b Have you noticed discharge/bleeding between periods?
    Not at all
    Occasional discharge/bleeding
    Substantial discharge/bleeding, occasionally
    Frequent discharge/bleeding between periods
    4.8 Have you noticed less discharge/bleeding when menstruating?
    Not at all
    Slightly less than normal
    Less than normal
    Menstruation has not occurred
    4.15 Have you experienced difficulty in reaching orgasm?
    Not at all
    Some difficulty
    More difficult than normal
    Rarely have orgasm
    4.16 Do you have problems with a dry vagina during intercourse?
    Not at all
    Some dryness
    More problems than normal
    Severe problems, must use lubrication
    Males only
    4.13 Have you experienced difficulty in reaching erection?
    Not at all
    Slightly more difficult than normal
    More difficult than normal
    Cannot get erection
    4.14a Have you experienced difficulties in ejaculation?
    Not at all
    Ejaculation slightly delayed
    Ejaculation delayed
    Cannot ejaculate
    4.14b Have you experienced early (premature) ejaculation?
    Not at all
    Ejaculation slightly early
    Ejaculation early
    Spontaneous ejaculations


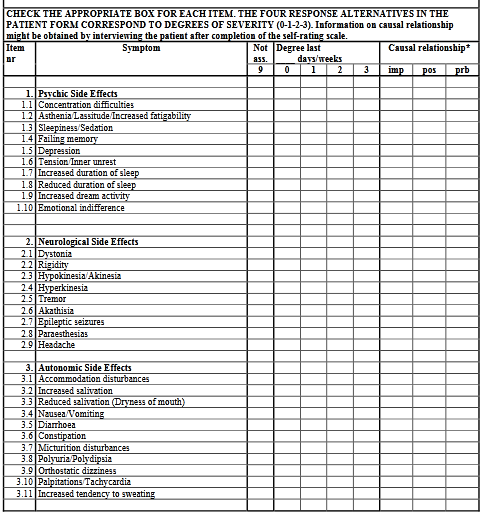


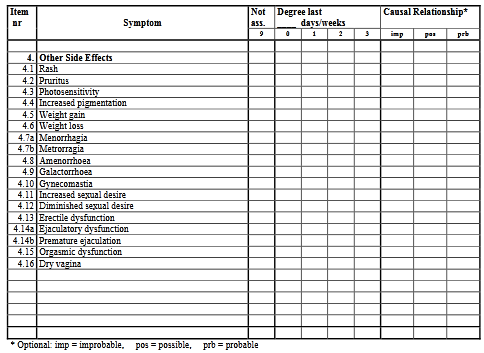


Lingjaerde O, Ahlfors UG, Bech P, et al. The UKU side effect rating scale. A new comprehensive rating scale for psychotropic drugs and a cross-sectional study of side effects in neuroleptic-treated patients. Acta Psychiatr Scand Suppl. 1987;334:1-100.

**SMARTS (Systematic Monitoring of Adverse events Related to TreatmentS) questionnaire**


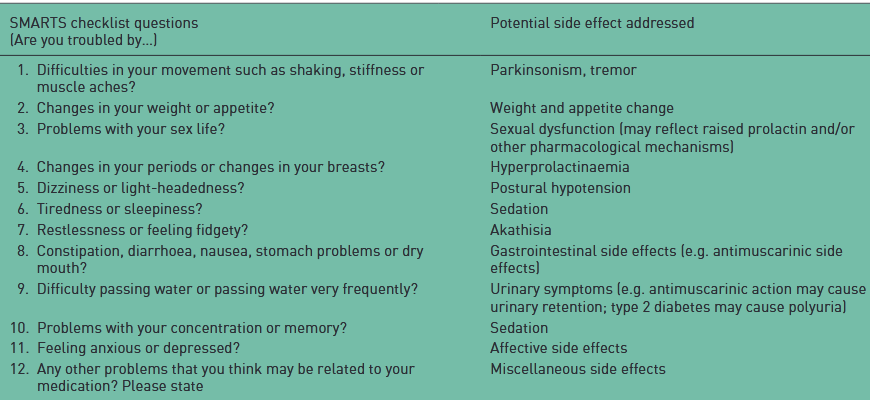


Haddad PM, Fleischhacker WW, Peuskens J, et al. SMARTS (Systematic Monitoring of Adverse events Related to TreatmentS): The development of a pragmatic patient-completed checklist to assess antipsychotic drug side effects. Ther Adv Psychopharmacol. 2014;4:15-21.

**EQ-5D-5L**


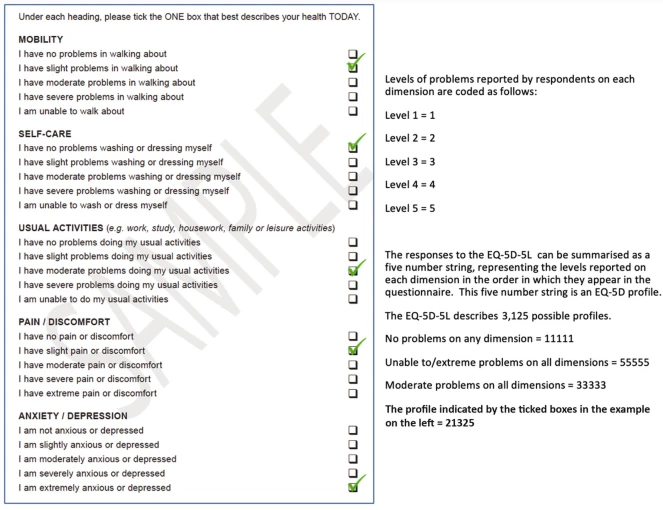


EQ-5D. Available at: <https://euroqol.org/eq-5d-instruments/eq-5d-5l-about/> (accessed on 17.02.2023)
